# Systematic Review of Metallic, Industrial, and Pharmaceutical Emerging Contaminants in Snow and Ice: A Global Perspective from Polar and High-Mountain Regions

**DOI:** 10.3390/molecules31050846

**Published:** 2026-03-03

**Authors:** Azzurra Spagnesi, Andrea Gambaro, Elena Barbaro, Jacopo Gabrieli, Carlo Barbante

**Affiliations:** 1 Department of Environmental Sciences, Informatics and Statistics, Ca’ Foscari University, 30172 Venice, Italyelena.barbaro@cnr.it (E.B.);; 2 Institute of Polar Sciences—National Research Council of Italy (ISP-CNR), 30172 Venice, Italy

**Keywords:** emerging contaminants, metals and metalloids, industrial compounds, pharmaceuticals, cryosphere, health and environmental impacts

## Abstract

Emerging contaminants (ECs) comprise diverse pollutant classes that are increasingly detected in remote environments due to their persistence and long-range transport potential. In cold regions, atmospheric cold-trapping processes favour their accumulation in high-altitude and high-latitude snow and ice, which act as sensitive archives and secondary sources of contamination. While previous studies have addressed individual environmental compartments (e.g., snowpack, glacier ice, meltwater), focusing on specific contaminant classes, a systematic review integrating the occurrence, behaviour and impacts of major EC groups in polar and alpine snow and ice is still lacking. To fill this gap, this work synthesised current knowledge on the environmental fate of three key EC categories in the cryosphere: metals and metalloids (MMs), industrial chemicals and by-products (ICBs), and pharmaceuticals and personal care products (PPCPs). PRISMA guidelines were accurately followed for research, which was based on a Google Scholar search combining keywords on cryospheric matrices (snow, firn, ice cores), geographical regions (Arctic, Antarctic, Alps, high mountains), and contaminant classes. Of 350 records initially identified, 300 met the eligibility criteria (post-industrial snow, firn, or ice cores studies) after excluding studies focused on aerosol or meltwater-only, method-focused papers, pre-industrial datasets, urban-only investigations, and duplicates. Risk of bias was qualitatively assessed through manual screening, evaluating matrix eligibility, temporal consistency, analytical methods, detection limits, and duplicate data, with particular attention to inconsistencies in ECs classification. Strict operational definitions were therefore applied to ensure methodological coherence. Concentration data were harmonised into a standardised database, and findings were synthesised through a structured narrative supported by tabulated datasets organised by matrix and site. Overall, the evidence indicates widespread occurrence of ECs in the global cryosphere, with spatial variability linked to emission sources, long-range transport pathways, and snow physicochemical properties. Climate-change-driven alterations of snow dynamics, glacier retreat and permafrost thaw are expected to modify partitioning equilibria and enhance the secondary release of legacy and contemporary contaminants. However, significant limitations persist, including geographical gaps, variability in analytical sensitivity, lack of long-term monitoring for certain EC classes, and inconsistencies in contaminant classification frameworks. Despite these constraints, the synthesis highlights consistent emerging patterns and underscores the need to strengthen existing environmental protocols to mitigate potential risks to ecosystems and human health.

## 1. Introduction

**Figure 1:**
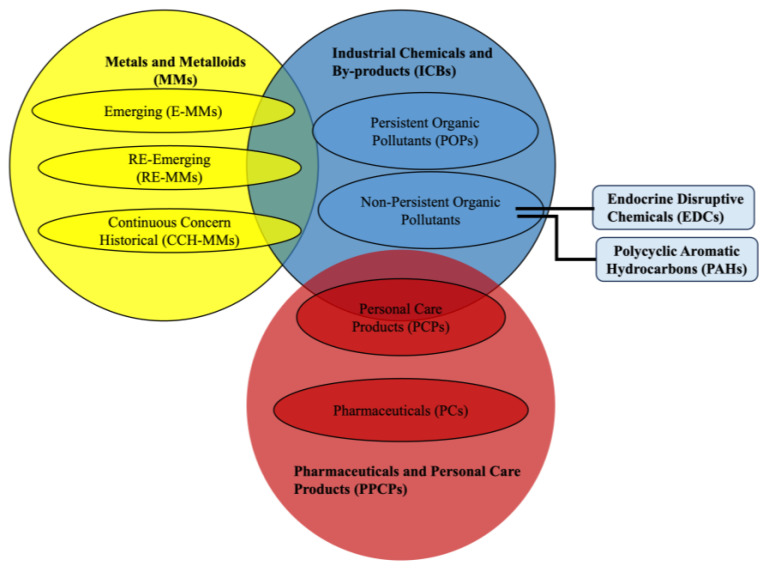
Venn diagram shows the three main categories of ECs discussed in this review, with relative sub-categories. Metals and Metalloids identified groups are Emerging (E-MMs), RE-Emerging (RE-MMs) and Continuous Concern Historical (CCH-MMs). The industrial Chemicals and By-products (ICBs) class includes Persistent Organic Pollutants (POPs) and Non-Persistent Organic Pollutants (Non-POPs). The latter group comprehends Endocrine Disruptive Chemicals (EDCs) and Polycyclic Aromatic Hydrocarbons (PAHs). Finally, the Pharmaceuticals and Personal Care Products (PPCPs) cluster includes Personal Care Products (PCPs) and Pharmaceuticals (PCs). Partial overlapping exists among the three main ECs categories discussed in this work, and they are represented by overlapping colours in the Venn diagram.

Emerging contaminants (ECs) are a diverse group of scarcely monitored and unregulated pollutants increasingly present in the environment, classified according to their origin, use, and chemical characteristics. Although numerous classification schemes for ECs have been proposed, including nanomaterials, microplastics, and other emerging groups, often with overlapping boundaries [1], a consistent framework that treats metals and metalloids (MMs) as new ECs and provides a global cryospheric evaluation of industrial chemicals and by-products (ICBs) and pharmaceutical and personal care products (PPCPs) is still lacking. These three main groups (Figure 1) span both the contaminants most frequently reported in the cryosphere (MMs, ICBs) and those for which evidence remains fragmentary (PPCPs). Notably, PPCPs are still scarcely investigated in cold regions, and their limited yet indicative detection in pilot studies underscores the need for targeted assessment.

These contaminants can enter the environment through various pathways, including leaching, atmospheric deposition, and discharge in wastewater [2]. Their ubiquitarian distribution, together with their ability to bioaccumulate in each environmental compartment, poses potential risks to ecosystems and human health, especially for populations who rely on glacial melt for drinking water and agriculture (e.g., [3]). Early evidence of these effects has recently been found at lower trophic organisation levels, but possible damaging effects are expected on macroinvertebrate communities for prolonged exposure to mixtures of ECs, and unknown cascade effects on the river food web [4]. These concerns highlight the need for urgent investigation and the development of global shared targeted policies [5].

Concurrently, recent advances in analytical techniques have expanded our ability to detect these compounds and to characterise their ecological and toxicological effects [6,7], stimulating research on their sources and patterns of environmental occurrence [8]. Despite this progress, substantial knowledge gaps remain regarding their interactions with environmental matrices and the mechanisms governing their fate and transport [6].

As research continues to map the pathways and behaviour of ECs across environmental compartments, the cryosphere stands out as one of the least explored yet most sensitive reservoirs of these contaminants. Affected by long-range transport (LRT) and cold-trapping mechanisms, these pollutants accumulate in polar and high-altitude regions, often far from their original sources [9]. They can later be remobilised and released into glacial freshwater systems, a process that is accelerated under ongoing climate warming, which can directly or indirectly affect the transport, pathways and environmental fate of persistent contaminants, making bioavailable those deposited in the past [10,11].

In the Arctic cryosphere, the most frequently detected ECs include ICBs like new persistent organic pollutants (POPs) (e.g., polybrominated diphenyl ethers (PBDEs) and organochlorine pesticides (OCPs)) (e.g., [12,13]) and long-recognised POPs, like per- and polyfluoroalkylic substances (PFAS) (e.g., [14,15]). Other relevant ICBs include brominated, chlorinated and organophosphate flame retardants (BFRs, CFRs, and OPEs) [16], and unintentionally produced polychlorinated biphenyls (uPCBs) (e.g., [17]). Moreover, personal care products (PCPs) and endocrine disruptive chemicals such as fragrances, UV filters and bisphenol A (BPA) [18] have been recently detected, while pharmaceutical compounds (PCs) have only been reported in a pilot study from [19]. Metals and metalloids, increasingly recognised as ECs in a broader sense due to their persistence in the environmental compartments, the bioaccumulation potential, and the growing scientific concern over their persistence, have also been found in the Arctic snow cover (e.g., [20]).

Similar patterns emerged in the Antarctic environment. Despite the protection guidelines in force, LRT processes and the increase in human activities in Antarctica are sources of many persistent contaminants not yet subject to regulatory criteria and often lacking standardised sampling and analytical procedures [10]. The localised environmental contamination particularly reflects the presence of ICBs, PPCPs, and MMs released by fuel combustion emissions, requiring urgent improvement in environmental monitoring and control protocols around scientific stations [21,22,23,24,25,26] to prevent the redistribution of ECs driven by snowpack and ice melting [10].

Beyond the polar regions, high-mountain environments play a crucial role in determining the environmental fate of these contaminants [27]. Evidence of contamination in alpine areas dates back to the early 1990s, when Calamari et al. (1991) [28] first proposed that high mountains could act as regional “cold traps” for POPs. This hypothesis was later confirmed by [29], who demonstrated that mountainous regions can serve as convergence zones for air masses and precipitation. In Europe, the proximity of densely populated and industrialised areas to the Alps enhances the deposition of contaminants that, although less persistent than classical POPs, are still capable of regional atmospheric transport over several hundred kilometres from their emission sources. This is particularly true for current used pesticides (CUPs) [30], whose presence have been recently reported in glacier meltwaters of the Italian Alps [31] and in glacial lakes of the Pyrenees [32], together with polycyclic musk fragrances. Although snow and ice compartments remain less explored compared to glacial and pro-glacial lakes in high-mountain regions, little evidence has demonstrated the presence of ECs even in these matrices. This is the case of PFAS, which are common additives in ski waxes for their water repellent properties, which have been detected in snow samples from family skiing areas at Alpine locations [33]. Similarly, metals and metalloids such as lead (Pb), mercury (Hg), and antimony (Sb), have been found in alpine glaciers, with concentration profiles tracing back to the early industrial revolution and continuing to the present day [34,35,36,37].

The contamination of snow and ice by emerging pollutants has also received growing attention in the Tibetan Plateau, which, together with the Arctic and Antarctica, comprises a major component of the Earth’s cryosphere [38]. In the Nam Co Basin, located in the central Tibetan Plateau, glacial ice, snow, and meltwater runoff have revealed detectable levels of PFAS with markedly higher concentrations in meltwater during the ablation season. Moreover, surface snow samples from the Eastern Tibetan Plateau revealed notable concentrations of metals like lead (Pb), gallium (Ga) and metalloids as arsenic (As) and antimony (Sb), employed for industrial products (e.g., batteries, metallic alloys, shielding for ionising radiations, dyes, additives, pesticides), which were found to be subjected to LRT from the surrounding densely populated areas of Asia [39].

Overall, this study offers a preliminary classification of the main groups of ECs, proposing a conceptual framework that may serve as a meaningful contribution and a valuable resource for the cryosphere research community. Nevertheless, given the current state of research and the comparatively greater availability of studies on metals and metalloids, this review naturally places more emphasis on these elements. Coverage of industrial organic contaminants and pharmaceuticals is comparatively more limited, reflecting existing knowledge gaps and highlighting priorities for future investigation.

## 2. Methods and Materials

**Figure 2:**
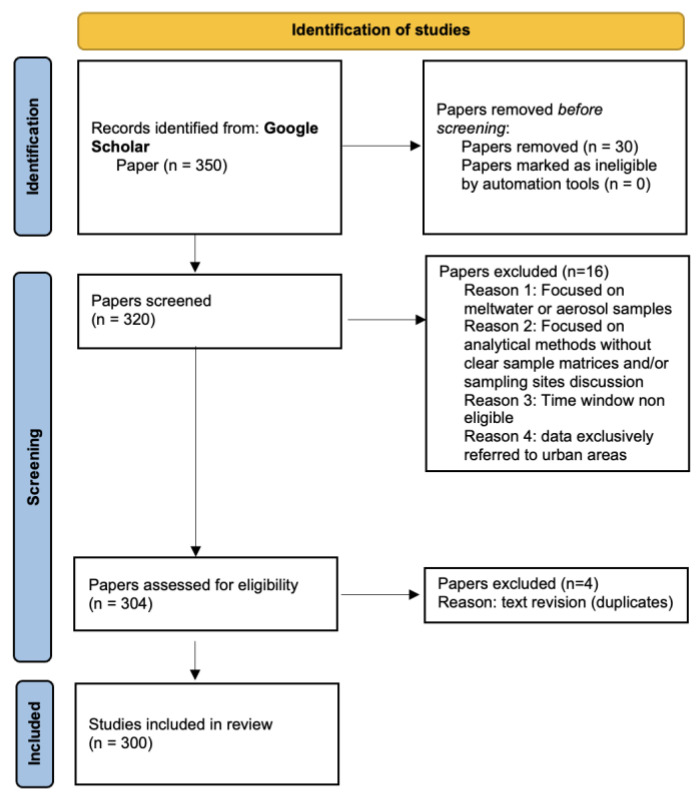
Simplified PRISMA flow diagram depicting the flow of information through the different phases of the review.

This systematic review was conducted in accordance with the PRISMA (Preferred Reporting Items for Systematic reviews and Meta-Analyses) 2020 guidelines, as schematically displayed in Figure 2.

**Figure 3:**
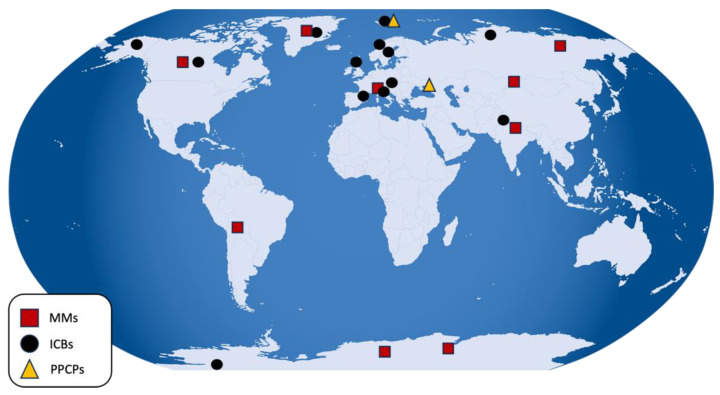
Geographic overview of sampling sites distributions. MMs, ICBs and PPCPs identify metals and metalloids, industrial chemicals and by-products, pharmaceuticals and personal care products, respectively.

The literature search was performed using the Google Scholar platform and included combinations of the following keywords: *snow*, *ice core*, *high mountain*, *Alps*, *Arctic*, *Greenland*, *Svalbard*, *Antarctica*, *metals and metalloids*, *lead*, *zinc*, *antimony*, *thallium*, *gallium*, *chromium*, *nickel*, *copper*, *cadmium*, *mercury*, *arsenic*, *review metals and metalloids snow ice*, *pharmaceuticals*, *personal care products*, *emerging contaminants*, as well as targeted expressions such as *review heavy metals snow ice* and *review emerging contaminants snow ice*. A date filter was applied to identify the most recent review articles, which served as the starting point for a broader exploration of primary studies addressing metals and emerging contaminants in the global cryosphere. The inclusion criteria comprised cryospheric matrices (snow, firn and ice cores) and a post-industrial revolution time window (particularly for heavy metal studies). Studies focusing exclusively on meltwater or aerosol samples were excluded. The selected papers were subsequently classified according to pollutant categories: metals and metalloids (MMs), industrial chemicals and by-products (ICBs), pharmaceuticals and personal care products (PPCPs). A further subdivision was made based on geographical reference sites to ensure a coherent narrative distinguishing between polar regions and high-mountain environments. The global distribution of all sampling sites included in this review is presented in Figure 3, which provides a comprehensive geographical overview of the investigated cryospheric regions. Both the study selection and data extraction processes were performed manually, without the use of artificial intelligence or automated screening tools. This approach was adopted to ensure full methodological control, conceptual consistency, and critical evaluation of heterogeneous datasets. Manual screening indeed enabled careful verification of matrix eligibility, accurate identification of post-industrial datasets within multi-century ice core records, critical evaluation of analytical methodologies and detection limits, and prevention of duplicate data extraction from review-derived primary datasets, ensuring scientific rigour in accordance with the PRISMA methodological framework. Given the heterogeneity in measurement units across studies, all concentration values were compiled into an Excel database and standardised to ensure comparability. Metals and metalloids were harmonised to µg L^−1^, whereas industrial chemicals and pharmaceuticals were converted to ng L^−1^ or reported in pg cm^−2^ yr^−1^ if fluxes were available, reflecting the most common adopted reporting units in the literature. A risk of bias issue emerged from inconsistencies in the classification of emerging contaminants. Categories adopted in the literature are not rigid, mutually exclusive, or universally standardised, and certain compounds may fall into multiple groups depending on the criteria applied. A representative example concerns polycyclic aromatic hydrocarbons (PAHs). Although some high-molecular-weight PAHs exhibit environmental persistence and long-range transport potential, the PAH class does not uniformly meet the defining criteria for Persistent Organic Pollutants (POPs) established under the Stockholm Convention on POPs. Therefore, to ensure regulatory alignment, methodological consistency, and reproducibility, this review adopted a strict operational definition of POPs limited to compounds formally recognised under international conventions. On this basis, PAHs were classified as Non-Persistent Organic Pollutants within the analytical structure of this work. To visually synthesise the results, Venn diagrams and bar charts were realised, complemented by comprehensive tables summarising the reviewed literature. These tables distinguish among cryospheric matrices (snow, firn, ice) and report measured concentrations for each compound with full accuracy and traceability.

## 3. Metals and Metalloids (MMs) in Polar and High-Mountain Regions

Anthropogenic metal and metalloid (MMs) contamination has impacted all environmental compartments since the industrial revolution, posing significant risks to both ecosystems and human health [40,41]. Metals and metalloids comprise a group of potentially toxic elements that have long been inaccurately grouped under the umbrella term “heavy metals” [42]. They are generally considered as historical and legacy pollutants, due to their widespread past use and the regulatory measures adopted [43]. However, ongoing technical transitions, expanding industrial applications, and climate-driven remobilisation processes (e.g., melting glaciers and thawing permafrost) emphasise their continuing evolving and environmental relevance. Given their toxicity at trace concentrations and their dynamic emission patterns, metals and metalloids (MMs) were classified in this review according to three operational criteria: (1) current emission dynamics (increasing primary emissions vs. legacy remobilisation), (2) expansion of technological or industrial applications in recent decades, and (3) degree of regulatory consolidation and scientific understanding of environmental fate and transport.

Based on these criteria, antimony (Sb), thallium (Tl), and gallium (Ga) can be classified as proper “emerging MMs” (E-MMs), reflecting their growing industrial use and the still limited understanding of their environmental behaviour. On the other hand, metals such as chromium (Cr), nickel (Ni), copper (Cu), zinc (Zn), cadmium (Cd), and mercury (Hg) can be considered as “re-emerging metals and metalloids” (RE-MMs), owing to their renewed applications, persistence, and high toxicity, with known environmental behaviour [44]. In this context, “re-emerging” denotes a resurgence of environmental concern despite established regulatory control and scientific knowledge.

Arsenic (As) and lead (Pb) have been extensively used since ancient civilisations [45], and can be classified instead as “historical continued concern metals and metalloids” (HCC-MMs). Their environmental presence is indeed predominantly linked to legacy contamination and long-term persistence rather than to expanding modern applications. Despite stringent regulatory restrictions, their high toxicity, bioaccumulation potential, and continuous remobilisation from historically contaminated soils, sediments, and industrial residues maintain their global significance [46,47,48,49,50]. In the absence of substantial renewed industrial expansion, As and Pb are not considered emerging or re-emerging, but rather persistent legacy pollutants with enduring environmental impact.

The presence of emerging, re-emerging, and continuous concern of historical metals and metalloids in snow and ice raises particular apprehension regarding their secondary release from the shrinking cryosphere under a changing climate. This process can increase their bioavailability and ecological risks in the freshwater systems, with potential cascading effects on neighbouring environments [2,46]. Therefore, these dynamics urgently demand multi-faceted regulatory approaches [9,26,41].

**Figure 4:**
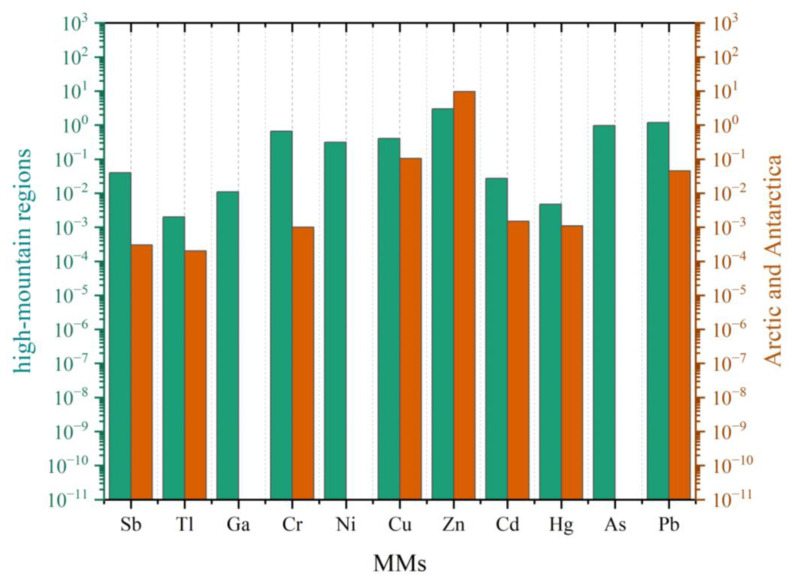
Bar chart of MMs concentrations in snow, firn, and ice sampled at high-mountain regions and poles. Concentrations are reported in logarithmic scales. Green bars represent concentrations retrieved at high-mountain regions, while polar sites concentrations are displayed in brown.

A comprehensive overview of detection sites, environmental matrices (i.e., snow, ice or firn), reference periods, average concentrations, and the corresponding literature sources for these pollutants is presented in Table 1, while a visual comparison of MMs concentrations in snow and ice from high-mountain regions and polar sites is displayed in Figure 4.

### 3.1. Emerging Metals and Metalloids (E-MMs)

#### 3.1.1. Antimony (Sb)

Antimony is a metalloid primary used as a flame retardant in consumer products, industrial materials, and specialised safety applications [80]. This sector accounts for most of its global consumption, while additional applications include metal alloys, pigments, plastics, and specialised industrial uses [81,82]. Antimony is ubiquitous in the environment, released into soils and aquatic systems as particulate fraction [83] through fossil fuel combustion [84], weathering of sulphide ores, leaching from mining wastes, and various anthropogenic activities such as smelting, metallurgical operations, and shooting [82]. In environmental systems, Sb predominantly occurs as Sb (III) and Sb (V), which influence its mobility and bioavailability [85].

In the cryosphere, Sb has been detected across high-altitude and polar regions, including the European Alps, the Tibetan Plateau, Everest, the Altai Mountains, and Antarctica [39,52,53,54,56,58,59,60]. Antimony concentrations in snow and ice typically remain below 0.1 µg L^−1^ (Table 1), with highest concentrations recorded in snow from the Tibetan Plateau during the 2017 snow season [39,53]. Compared with highly bioaccumulative metals such as Hg and Cd, Sb is generally detected at lower concentrations in cryospheric matrices, although it exhibits comparable patterns of LRAT and glacial storage.

The potential of Sb for bioaccumulation and biomagnification through food webs, and its remobilisation capacity from glacial archives under ongoing climate warming collectively raise increasing concern within the scientific community, as glaciers represent a secondary source of contamination for downstream ecosystems.

Due to its toxicity and extensive use, Sb is classified as a priority pollutant by the European Union and the U.S. Environmental Protection Agency (EPA) [80]. Indeed, the 36 mg kg^−1^ in soil and 20 μg L^−1^ in drinking water thresholds set by the World Health Organization (WHO) [86] are frequently exceeded in regions affected by mining, smelting, and other industrial processes [87,88]. Elevated Sb levels threaten ecosystem integrity, agricultural productivity, and water quality [89,90], posing potential risks to human health, including hepatic, respiratory, and dermatological toxicity [80,91]. However, it remains unclear what mechanism is responsible for antimony’s genotoxicity [92]. Thus, further research is required to clarify its distribution in multiple ecological environments and better assess its potential risks [69,93].

#### 3.1.2. Thallium (Tl)

Thallium is a rare but widely dispersed element found in various raw materials and released through anthropogenic activities, including metal refining, waste incineration, and fossil fuel combustion [94]. Owing to its high toxicity relative to several other trace metals (e.g., Hg, Cd, and Pb) [95], Tl is recognised as a priority pollutant in several countries, including China, the U.S., and Canada [96]. However, its environmental distribution, geochemical cycling, and ecological risks in atmospheric particles remain poorly understood, which may pose significantly hidden risks due to long-term exposure. This is partly due to its low crustal abundance, which makes its quantification difficult using cost-effective and widely available analytical methods, and partly because tools for assessing the ecological risk of Tl to surrounding ecosystems are still underdeveloped [93].

Within cryospheric environments, Tl has been sporadically detected in snow and ice at concentrations typically below 0.01 µg L^−1^ (Table 1). Records exist from snow and ice samples collected in North America [61], Greenland [63], and high-mountain regions of Asia [59,62], spanning the post-industrial revolution period to the early 21st century. The presence of Tl in these remote environments suggests atmospheric LRT and deposition from anthropogenic sources, notably coal combustion and metallurgical emissions (e.g., [59,61]). Although data remain scarce, its detection in polar and alpine snow indicates that Tl may follow similar pathways of global dispersion as other metallic emerging contaminants. However, compared to more extensively studied metals such as Pb, the cryospheric dataset for Tl remains sparse, limiting robust regional comparisons.

Once deposited and incorporated into glacial layers it can be later released during melting events, potentially contributing to secondary contamination of meltwater systems and downstream ecosystems.

In non-cryospheric settings, elevated Tl concentrations have been reported in groundwaters [93], nearly abandoned mining districts like Molini di Sant’Anna (5–37 µg L^−1^, [97]) or Valdicastello Carducci village (4.3–27.8 µg L^−1^, [98]), in the Apuan Alps (Italy). Thallium can be accumulated in bones, renal medulla, and in the central nervous systems of humans, once absorbed through mucous membranes [94]. Therefore, addressing the pressing issue of atmospheric Tl pollution and take proactive measures to resolve it appears particularly crucial.

#### 3.1.3. Gallium (Ga)

Gallium is a trace metal with an average crustal abundance of 17 ppm [99]. It is never found in its native state and typically occurs as a minor constituent in aluminium, zinc, and iron ores, as well as in coals and bauxite deposits [100]. Gallium is classified as a technological critical element (TCE) due to its increasing demand and limited recyclability, which raise growing environmental concerns, particularly given its extensive use in the form of Gallium arsenide (GaAs) and Gallium nitride (GaN), which are recognised as ecotoxic forms [101]. Gallium arsenide and GaN are employed for transistors and light-emitting diodes (LEDs) production [102]. These materials are increasingly used in a wide range of electronic devices and optoelectronic technologies [101]. As Ga remains largely irreplaceable in many modern technological applications, its global demand is projected to increase more than 20-fold by 2030 compared to a production yield of 275 tons in 2012 [103]. Gallium is also used in dental materials and for treatment of cancer [104], as it does not accumulate in biological tissue and functions as an iron mimetic, perturbing iron-dependent proliferation processes in cancer cells [105]. However, Ga can easily diffuse in soils, sediments, and groundwaters [106], altering the local chemistry and potentially interfering with essential micronutrients such as iron and aluminium, causing metabolic disfunctions in microorganisms, vegetation and mammalian cells [105].

Therefore, growing concern has emerged over the increasing demand and use of Ga, which inevitably contributes to its presence even in remote regions such as the polar areas and high-altitude glaciers, where concentrations ranging from 0.002 µg L^−1^ in the Western Siberian Lowland [56] to 0.02 µg L^−1^ in Canada [61] have been reported (Table 1). Although available data are limited in both temporal coverage (2005–2014) and geographic scope, the detection of Ga in high-latitude and high-altitude snow indicates potential long-range atmospheric transport of this element. In contrast to legacy metals and metalloids such as Pb and As, Ga represents an emerging technology-driven contaminant whose cryospheric occurrence is only beginning to be documented.

### 3.2. Re-Emerging Metals and Metalloids (RE-MMs)

#### 3.2.1. Chromium (Cr)

Chromium is a transition metal that occurs naturally in rocks, volcanic emissions, water, and soil in two primary forms: the hexavalent (Cr (VI)), highly toxic and soluble in water, and the trivalent (Cr (III)), less toxic form [107,108]. Hexavalent chromium (Cr VI) can accumulate in the human body via inhalation, ingestion, or contaminated food and water, raising increasing concern due to its carcinogenic risk [109]. Despite its natural presence, the majority of environmental Cr contamination originates from anthropogenic activities. In particular, metallurgical, chemical, and refractory industries represent the principal industrial sources of atmospheric Cr emissions [110]. Chromium is extensively used in metallurgy and chemical industries, particularly in stainless steel production, surface treatments, and pigment manufacturing. These activities account for the predominantly anthropogenic origin of environmental Cr contamination.

In the cryosphere, Cr has been found at concentrations varying from 0.001 µg L^−1^ in Antarctic snow for the period 1983–1990, to 10 µg L^−1^ at the Northeastern Tibetan Plateau in 2017 sampled snow [53]. Higher concentrations recorded at high-altitude glaciers compared to those found at the poles are mainly attributed to the combustion of fossil fuels, emissions from iron and steel plants, and waste burning and disposal of solid waste.

#### 3.2.2. Nickel (Ni)

Nickel is a transition metal naturally present in the Earth crust, typically bound to oxygen and sulphur as oxides and sulphides. Owing to its unique physical and chemical properties (e.g., corrosion resistance, catalytic activity, and magnetic properties), Ni is widely used in metallurgy and modern industry, such as for alloy production, electroplating, batteries, and various industrial and catalytic applications (e.g., [111]). The extensive use of Ni-containing products, combined with emissions from fossil fuels, waste incineration, and wood combustion [112], inevitably leads to environmental contamination at all stages of production, recycling, and disposal [113]. Although Ni is an essential micronutrient for plants, contributing to their defence against pathogens and herbivorous insects [114], as well as for animals and soil microbes’ growth [112], excessive concentrations can be toxic, with effects depending on exposure pathways and the solubility of Ni compounds [113]. Nickel exhibits limited mobility under neutral to alkaline soils and reducing conditions, which increases in acidic, organic-rich soils, where it may pose a risk to groundwater quality. Toxicity largely results from interference with the metabolism of essential metals, such as iron (Fe), manganese (Mn), calcium (Ca), zinc (Zn), copper (Cu) or magnesium (Mg), which can suppress or modify normal biochemical and physiological processes [111].

Growing concern derives from nickel’s potential release, accumulation, and biochemical interference with competing cations in meltwater systems, despite low concentrations (between 0.1 and 1.6 µg L^−1^) have been detected so far in snow and ice from high-altitude glaciers (i.e., Alps, Tibetan Plateau). Notably, the highest concentrations of Ni have been revealed in the 2017 snowfall sampling at the NorthEastern Tibetan Plateau, reaching 1.6 µg L^−1^. Notably, the Miaoergou Glacier in the eastern Tianshan Mountains revealed the highest concentration of this element compared to the other investigated glaciers (Laohugou, Yuzhufeng, and Qiyi glaciers), likely derived from fuel burning and traffic emissions transported from nearby regions [53].

#### 3.2.3. Copper (Cu)

Copper is a transition metal essential to life [115] that occurs in nature in the form of sulphide and oxide ores, salt minerals and as a native element [116]. It is the second most used non-ferrous metal in industry [117], although copper dominance has challenged in recent years by aluminium for radiators and fibre optics in telecommunications. Nevertheless, this versatile metal can still be found in multiple industrial and residential uses thanks to its excellent electrical and thermal conductivity, corrosion resistance, and ductility [118]. Copper is widely used in electrical, electronic, construction, and renewable energy technologies due to its high conductivity and corrosion resistance (e.g., [119,120]). While Cu is an essential micronutrient, elevated concentrations may pose ecological risks [121,122] reaching water bodies and passing through the food chain by incorporation into fishes and other aquatic organisms [123].

The presence of Cu in snow and ice from remote high-latitude and high-altitude regions, ranging from 0.2 µg L^−1^ in Antarctica [69] to 2.3 µg L^−1^ in ice cores from Bolivia [67], is of ongoing concern, as it reflects the LRT of anthropogenic emissions and highlights the potential for environmental accumulation and remobilisation in sensitive cryospheric ecosystems. Although average Cu concentrations in snow and ice from high-altitude glaciers are around 0.4 µg L^−1^ in the 21st century, a peak value of 2.3 µg L^−1^ was recorded in 1988 at the Sajama ice cap (Bolivia), likely associated with metal production activities in South America.

#### 3.2.4. Zinc (Zn)

Zinc is a transition and widely distributed non-ferrous metal, notable for its resistance to corrosion in the presence of reactive substances, salt water, fresh water and atmosphere [124], as well as its important roles in biology and public health [125]. The major uses of zinc metal are in galvanising iron and steel against corrosion, producing brasses and alloys for die casting, and serving as key component in the chemical industry for rubber, ceramics, and pharmaceuticals. Emerging technological and biomedical applications are further increasing global Zn demand (e.g., [126,127,128]).

Zn has been detected in snow and ice cores from high-altitude glaciers at concentrations ranging from 1 to 10 µg L^−1^ since the 1960s (Table 1), while lower concentrations (<0.1 µg L^−1^) were recorded in Greenland snow and ice from 1983 to 1984 [70]. Unexpectedly, Antarctic ice, about 75 km south of the Chinese Zhongshan station, exhibited the highest average Zn concentration (19.2 µg L^−1^) over 1968–2016, representing a marked increase from pre-1968 period. This elevated Zn concentration in Antarctica was attributed primarily to non-ferrous metal mining and smelting in South America, Southern Africa and Australia [66,69]. However, the temporal pattern also correlates with the Australian scientific expeditions in this region, and the intensified human activities by Australia, Russia, and China [69]. In the framework of the ongoing climate change, thus considering the potential secondary release from melting glaciers and thawing permafrost of high concentrations of Zn, elevated exposures may pose risks to human- and ecosystem health [25].

#### 3.2.5. Cadmium (Cd)

Cadmium is a flammable metal with very low solubility in water and a high resistance to corrosion, which makes it valuable for coating more reactive metals to prevent oxidation. Its anthropogenic emissions primarily originate from the mining and processing of Cd-bearing sulphide ores, particularly those of zinc, copper, and lead. Processed Cd is widely used in the manufacture of pigments, Ni-Cd batteries, metal coatings, stabilisers, and various alloys. Globally, human activities are estimated to release between 5000 and 13,000 tons of Cd into the environment each year, and as Cd cannot be degraded, its concentration in the environment tend to increase steadily [129]. Cadmium contaminated areas can act as continuous sources of airborne Cd dust, which can be transported over long distances by wind. In aquatic environments, Cd contamination arises mainly from mining effluents, battery recycling, waste incineration, fossil fuel combustion, and the use of phosphate fertilisers. Once introduced into water bodies, Cd can be readily absorbed and accumulated by aquatic organisms, leading to biomagnification through the food web [130]. In addition, the relatively high Cd content in phosphate-based fertiliser has been shown to inhibit seed germination and impair crop growth, suggesting that excessive fertiliser application can further increase Cd levels in soils and, finally, in the food chain [107]. Moreover, Cd is considered one of the most toxic metals, as it may exert a wide range of toxic effects via a variety of mutually interconnected mechanisms. The major mechanisms and molecular pathways of Cd toxicity involve the gene regulation, oxidative stress, mitochondrial apoptosis, autophagy, and interactions with essential metals [131].

Significant enrichments of Cd, relative to the natural levels, detected in the most recent Greenland snow [71] and high-mountain regions in Canada [61], Alpine sector [34,52], and Tibetan Plateau (e.g., [53,54]), have been attributed to anthropogenic inputs, with concentrations ranging from 0.001 to 0.13 µg L^−1^ since the end of the 20th century (Table 1). Among the metals reviewed, Cd shows one of the clearest links between industrial emissions and sustained cryospheric enrichment, particularly in rapidly industrialising regions. Indeed, although the emissions of Cd should have continuously decreased since the mid-1970s, in line with the improvements of pollution reduction technology, particularly in Europe and North America [132], the emissions of Cd in China rapidly increased from ~100 t in 1980 to ~800 t in 2010, in line with the rapid increase in energy consumption and industrial production [133], resulting in the negligible decline of Cd concentration levels in snow between the 1980s and 2000s. Therefore, over the last 15 years, the Comprehensive Environmental Response, Compensation, and Liability Act (CERCLA) has permanently listed Cd in its priority list of hazardous materials [134], given its recognised carcinogenicity and toxicity even at low doses (e.g., [135]).

#### 3.2.6. Mercury (Hg)

Mercury exists in several chemical forms, ranging from elemental Hg (Hg^0^) to inorganic and organic Hg compounds, with oxidation states +1 or +2 [136], each posing different risks to the natural environment and human health. Elemental mercury, which is liquid at room temperature, readily evaporates into toxic vapours, while inorganic Hg occurs naturally in minerals or as impurities and is released mainly from coal combustion and industrial effluents. Once emitted, Hg undergoes complex biogeochemical transformations, forming organic derivatives such as methylmercury (MeHg), a highly neurotoxic compound that bioaccumulates and biomagnifies through aquatic food webs [107,137]. Unlike most other metals discussed, Hg undergoes efficient biomethylation, which enhances its bioaccumulation and biomagnification potential in aquatic food webs.

Mercury is recognised as one of the most toxic pollutants, released into the environment from both natural and anthropogenic sources. However, anthropogenic emissions account for nearly two thirds of total Hg emissions, primarily through fossil fuel combustion, metal mining, ore processing, cement and gold production [137]. Furthermore, with the rapid progress of nanoscience, Hg has recently found new application in the production of mercury–oxide–zinc batteries operating in alkaline environments [138]. Developing countries, particularly in Asia, contribute substantially to global Hg pollution due to limited emission controls, with China currently representing the largest atmospheric source as a result of its rapid industrial and urban growth [139,140].

Concentrations of Hg^2+^ within the global cryosphere are provided by few examples in the literature, which report concentration ranging from 0.0004 µg L^−1^ revealed in snow/ice core collected in 1990 from Col du Dôme on the Mt Blanc, France [37], to 0.04 µg L^−1^ in snow sampled in 2005–2010 from the central Tibetan Plateau [73]. This hundred-fold increase in concentrations found in snow from the central Tibetan Plateau compared to the Alpine record seems to reflect the substantial contribute of Asian countries to Hg emissions (e.g., [141]). Despite the Minamata Convention has limited the Hg release to 15 mg kg^−1^ in contaminated waste [142], the environmental cycle of Hg remains active, contributing to re-emissions. Therefore, Hg pollution continues to be one of the most important environmental urgencies [143].

### 3.3. Continued-Concern Historical Metals and Metalloids (CCH-MMs): Arsenic (As) and Lead (Pb)

#### 3.3.1. Arsenic (As)

Arsenic remains one of the most hazardous agents worldwide, ranking first in the U.S. CERCLA Priority List of Hazardous Substances [134]. It typically occurs in natural waters as arsenite (As(III)) and arsenate (As(V)) ions, whose high solubility facilitates widespread transport in aquatic systems. The complex chemistry and potential toxicity of As, combined with its extensive historical use as a pesticide, chemotherapeutic agent, and ingredient in consumer products [144,145], make it a persistent subject of environmental and toxicological concern. Nowadays, nearly 106 countries are affected by groundwater As contamination and an estimated 230 million individuals worldwide are exposed to its adverse health effects, ranging from gastrointestinal distress to carcinogenesis, particularly affecting the skin, bladder, and lungs. Given the severity of As contamination, the maximum permissible level of arsenic in drinking water has been established at 10 µg L^−1^ by WHO, EPA and European Union (EU) [46].

Within the cryosphere, arsenic behaves as LRT metalloid, primarily introduced through atmospheric deposition of fine particles originating from coal combustion and smelting activities [146]. Snow and ice thus act as temporary sinks, trapping both particulate and dissolved forms of As that can later be released during seasonal melting. Although available data are still limited, existing studies indicate As concentrations ranging from 0.2 µg L^−1^ in the Western Siberian Lowland [56] to 3 µg L^−1^ in Bolivian glaciers [67]. These findings suggest that atmospheric deposition and cryospheric remobilisation may represent a non-negligible pathway for arsenic input into high-altitude and polar aquatic systems. The potential bioavailability of As in glacial meltwaters, particularly under accelerated deglaciation, raises growing concern about secondary contamination of pristine environments and downstream ecosystems.

#### 3.3.2. Lead (Pb)

Lead is a naturally occurring element present in trace amounts within the earth’s crust, and the second most toxic metal released by industrial activities [47]. Historically, Pb has played a central role in technological and industrial development, being extensively used in plumbing, construction, batteries, alloys, gasoline additives, paints, and electrical appliances [147]. Despite its widespread applications, Pb is a potent neurotoxin with severe and well-documented adverse effects on human health [148]. Due to its non-biodegradable nature and its accumulation potential [149], Pb can enter the human body through multiple exposure pathways, including direct contact, air, water, and soil, inducing oxidative stress, and damaging lungs, liver, brain, reproductive and nervous system, depending on exposure levels [150].

In the cryosphere, Pb serves as a clear indicator of long-range atmospheric transport and global anthropogenic activity. Historical ice-core records from polar and alpine glaciers reveal sharp increases in Pb deposition corresponding to the industrial era, particularly during the widespread use of leaded gasoline [151]. Following the Clean Air Act, which banned the uses of Pb in gasoline from the 1970s [152], Pb concentrations in snow and ice drastically declined worldwide, from values up to 7.7 µg L^−1^ recorded in Mont Blanc ice during the 1960s to current levels of 0.1–0.7 µg L^−1^ detected in snow from Canada and the Tibetan Plateau (Table 1). Lead therefore represents a well-documented case of regulatory success in reducing cryospheric contamination, contrasting with the more recent and less controlled trends observed for emerging metals. Nevertheless, Pb remains detectable in recent snowfall depositions, reflecting its atmospheric persistence. Therefore, its potential remobilisation through glacial meltwater can represent a secondary source of contamination for remote aquatic ecosystems and endangers human populations who rely on glacial meltwater for drinking water and agriculture [9].

Across the discussed metals and metalloids, common patterns emerge: LRT, glacial accumulation and potential remobilisation under warming conditions. However, their relative toxicity, mobility, and regulatory thresholds differ substantially, underscoring the importance of comparative assessments across contaminant classes and regions.

## 4. Industrial Chemicals and By-Products (ICBs) in Cryospheric Environments

**Figure 5:**
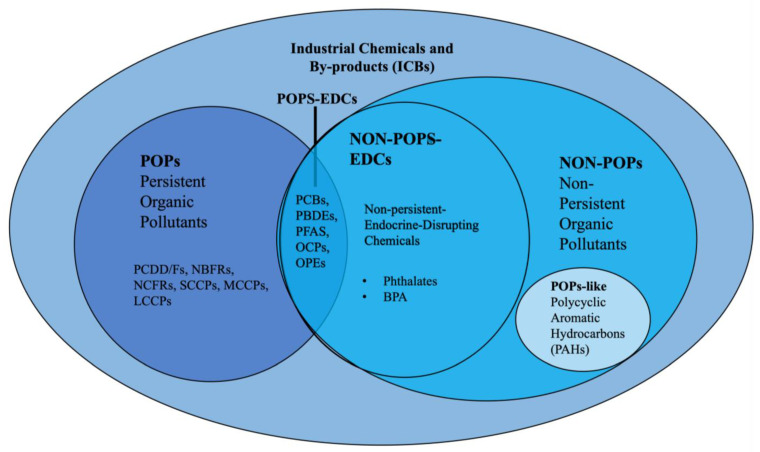
Venn diagram. Industrial contaminants found in the cryosphere: distinction between POPs and non-POPs classes. Within the POPs cluster, the following species were identified: Dioxins and Furans (PCDD/Fs), Novel Brominated and Chlorinated Flame Retardants (NBFRs, NCFRs), and short, medium, and long-chain Chlorinated Paraffins (SCCPs, MCCPs, LCCPs). The overlapping POPs-EDCs group comprehends instead Polychlorinated Biphenyls (PCBs), Polybrominated Diphenyl Ethers (PBDEs), Per- and poly-fluoroalkyl substances (PFAS), Organochlorine Pesticides (OCPs), and Organophosphate Esters (OPEs). Non-POPs-EDCs includes phthalates and Bisphenol A. Within the Non-POPs class, the POPs-like group is identified, characterised by the presence of Polycyclic Aromatic Hydrocarbons (PAHs).

The emerging industrial contaminants released from major industrial sectors such as textiles, leather, rubber, cement, and chemical manufacturing encompass a wide spectrum of substances with diverse structures and functions, collectively termed as “emerging” because they were historically excluded from routine monitoring and regulatory frameworks [7]. These pollutants can be distinguished into two main groups according to their environmental behaviour: persistent organic pollutants (POPs) and non-persistent pollutants (NON-POPs), the latter including endocrine disruptive chemicals (EDCs) and POPs-like compounds such as Polycyclic Aromatic Hydrocarbons (PAHs) [153]. Differences in their physiochemical properties influence climate-driven patterns of LRT and accumulation. However, these classifications are not rigid, mutually exclusive and universally standardised, as some compounds may belong to multiple categories depending on the adopted criteria (Figure 5). In some studies, PAHs are classified as POPs because of their persistence, bioaccumulative potential, and toxicity (e.g., [154]). In others, however, they are treated as a distinct group, as only high molecular weight PAHs exhibit substantial environmental persistence and not all PAHs show bioaccumulative potential (e.g., [155]). This variability highlights that the simple distinction between POPs and non-POPs is largely operational and context-dependent.

### 4.1. Persistent Organic Pollutants (POPs)

Persistent Organic Pollutants (POPs) are carbon-based chemicals characterised by high persistence, bioaccumulation potential, and ability to undergo LRT [156,157,158]. This group includes dioxins and furans (PCDD/Fs), chlorinated paraffins of short, medium- and long chain lengths (SCCPs, MCCPs, LCCPs), and novel brominated and chlorinated flame retardants (NBFRs, NCFRs) (Figure 5) [159]. They originate from agriculture, industry, combustion processes and chemical manufacturing, and are capable of dispersing far beyond their emission sources [160]. Their low molecular weight and semi-volatile conditions favour indeed their atmospheric LRT driven by global distillation (a.k.a. “grasshopper effect”), a process involving cycles of volatilisation, condensation, and re-emission that enables their progressive movement toward polar regions [161]. In contrast, at middle and low latitudes, POPs are primarily influenced by seasonal evaporation and diurnal temperature changes [162]. Atmospheric concentrations are shaped by two climate-sensitive, opposing processes: chemical degradation and volatilisation, both expected to intensify under rising temperatures [163]. Upon reaching high-latitude and high-altitude environments, POPs are efficiently removed from the atmosphere by wet and dry depositions. Particularly, snowfall serves as one of the most effective mechanisms for removing atmospheric organic pollutants [164], a process that depends on the physiochemical properties of POPs and the surface area of snow [165]. This leads to their accumulation in snowpacks and glacier ice through cold-trapping mechanisms [166]. Seasonal snow metamorphism and compaction further concentrate these contaminants, delaying their release until melt onset. During spring and summer, snowmelt, glacier ablation and permafrost thaw, currently accelerated by climate warming, become major pathways transferring stored POPs into aquatic and coastal ecosystems [16,160,162,167]. Evidence of this accelerated secondary release to downstream lakes, rivers, oceans and marine food webs has been documented across polar and mountain regions, including the Canadian Rockies [168], the European Alps [169], the Tibetan Plateau [170], and multiple Arctic sites such as Greenland fjords [171], Svalbard [172], Ellesmere Island [173], and Alaska [174]. Re-volatilisation from glacier surfaces represents another pathway for the release of POPs. This process has been estimated to contribute up to 60% to the deposition of chlorinated compounds [175]. As POPs accumulate in the atmosphere, they eventually return to glacier surfaces via wet deposition, creating a cyclical process [162].

Increasing human activity in the Arctic (e.g., shipping, tourism, fisheries, and resource development) is also projected to enhance the potential for local emissions of these toxic compounds [15]. Therefore, regulatory measures remain crucial. The Stockholm Convention, which initially restricted 12 POPs mainly from agricultural and industrial applications, has progressively expanded its list to 34 globally regulated substances as of 2024, with additional chemicals currently under evaluation [176]. Nevertheless, POPs continue to be detected in the cryosphere at critical concentrations, underscoring the need for sustained monitoring and assessment. Table 2 summarises their presence in the cryosphere, with relative concentrations (ng L^−1^) and/or fluxes (pg cm^−2^ yr^−1^). Novel brominated and chlorinated flame retardants are discussed in the text but are not included in Table 2 because their presence in snow and ice from remote environments has not yet been documented in the literature. However, they have been reported in aerosols from Arctic, Antarctic, and high-altitude regions.

Among POPs, **Dioxins and furans (PCDD/Fs)** are a class of tricyclic compounds with a total of 210 species, including 75 chlorinated diphenyl dioxins and 135 chlorinated dibenzofurans [179]. These classes share chemical similarities and biological characteristics [161]. Among them, 30 dioxin-like species belong to closely related groups, including polychlorinated dibenzo-p-dioxins (PCDDs), polychlorinated dibenzofurans (PCDFs), and certain PCBs. Polychlorinated dibenzo-p-dioxins and polychlorinated dibenzofurans are unintentional by-products of industrial syntheses, combustion processes, and chlorine bleaching, but they can also form naturally through wildfires and volcanic eruptions [180,181]. Their persistence, LRT, bioaccumulation, and toxicity enable them to impact ecosystems far beyond emission sources, including remote polar regions [182,183,184]. Although global anthropogenic PCDD/Fs emissions decreased from 48.8 kg TEQ in 2002 to 36.2 kg TEQ in 2018 [185], this decline is partly counterbalanced by rising natural emissions (i.e., biomass burning), which are sources expected to intensify due to global warming [182]. Nevertheless, to date, PCDD/Fs in the cryosphere have been reported only in Antarctic surface snow [177]. Among the 17 priority PCDD/Fs congeners, only 1,2,3,4,6,7,8-heptachlorodibenzo-p-dioxin (HpCDD) and octachlorodibenzo-p-dioxin (OCDD) were detected, at concentrations of 0.00013 ng L^−1^ and 0.0003–0.0005 ng L^−1^, respectively. These findings are consistent with aerosol samples collected in 2009–2010 at Faraglione Camp, Northern Victoria Land, Antarctica, where LRAT was suggested as a primary contamination source [186]. Nevertheless, HpCDD and OCDD may also originate from local Antarctic sources, as they were the most frequently detected congeners in ambient air at McMurdo Station [187].

**Novel brominated and chlorinated flame retardants (NBFRs, NCFRs)** are a large group of chemicals used in furniture, plastic, textiles, foams, and electronic devices to delay or prevent flaming in place of former banned flame retardants [188]. They are relatively new on the market and constitute approximately 25% of all commercially used flame retardants [161]. Given their high mobility and LRT potential, these substances have been recently detected in Antarctic aerosol [189], in the Arctic [190], and evidence of NBFRs have been recently reported for five mountain valleys in the Himalayas [191]. Moreover, bioaccumulation of NBFRs in marine food webs has recently been investigated at the poles [192], confirming the widespread presence of these contaminants in polar ecosystems. However, NBFRs/NCFRs have not been quantified in snow and ice so far. Nevertheless, the occurrence, distribution patterns, and ecological risks of brominated flame retardants have been recently investigated in Northeast China within multiple wetland compartments, including water, sediment and soil, demonstrating that ice can act as a temporary sink for several NBFRs and may pose moderate ecological risks [193].

**Chlorinated paraffins (SCCPs, MCCPs, LCCPs)** are a class of chlorinated n-alkanes widely used in leather industry, adhesives, rubber, paints and as secondary plasticisers in flexible polyvinyl chloride (PVC) [194]. These substances, classified into three groups according to their carbon chain length, as short-chain (C10-C13, SCCPs), medium-chain (C14-C17, MCCPs), and long-chain (C > 17, LCCPs) CPs [194], and listed in 2017 as POPs under the Stockholm Convention in Annex A (elimination) [195], have been widely detected in environmental matrices. Nevertheless, evidence of CPs in snow and ice remains extremely limited in the literature. To date, the only documented detections involve short-chained and medium-chained CPs measured in two urban snow deposit samples, at concentrations of 330 and 32,000 ng L^−1^, respectively [178].

### 4.2. Non-Persistent Organic Endocrine Disruptive Chemicals (Non-POPs EDCs)

Non-persistent endocrine-disrupting chemicals (EDCs) are exogenous substance or mixture that alters functions of the endocrine system causing adverse effects in intact organisms or its progeny and subpopulations [196,197]. This class of substances is primarily defined by their biological mode of action rather than by environmental persistence and bioaccumulative potential.

The list of substances characterised as EDCs is continually increasing, and although substantial scientific progress has been made in the field, public concern remains high due to constant consumer exposure and evidence linking EDCs exposure and human diseases [198]. Despite recent scientific efforts to advance the understanding of environmental dynamics of endocrine disruptive compounds, research remains limited due to their relatively recent identification [19]. In the EDC class, non-persistent industrial endocrine disruptors like phthalates and bisphenol A are included (Table 3). For the scope of this review, only these non-persistent pollutants have been considered within the EDC class. Chemicals with borderline persistence have instead been categorised under the POPs-EDCs group.

**Phthalates** are a group of compounds widely used in cosmetics, personal care products, pharmaceuticals, medical devices, children’s toys, glow sticks, lubricant, waxes, food packaging, insecticides, and cleaning and building materials [204]. In addition, these compounds are employed as plasticisers in PVC plastics. Among plasticisers, the most warring and widely used are di-(2-ethylexyl) phthalates (DEHPs) [205] largely employed worldwide up to the early 2000s. However, these compounds are not chemically bound to PVC, so that they can leach, migrate or evaporate into indoor air and atmosphere, finally diffusing in all the environmental compartments. Consumer products containing phthalates can result in human exposure through direct contact and use, and indirectly through leaching in other products or general environmental contamination, causing adverse effects in the liver, kidney and testes [206]. Moreover, phthalates can alter thyroid gland tissue functioning; therefore, they have been classified as suspected endocrine disruptors in humans, as they can bind to nuclear hormone receptors and can mimic or influence the effect of physiological hormones introduced into the human body [207]. Their presence in the Arctic cryosphere has been mostly detected in snow samples from the Russian Arctic archipelagos, at concentrations between 10 and 300 ng L^−1^ [199,200]. Phthalates were also found in Antarctic snow collected from seven stations, including Wood Bay, Vegetation Island, Mount Melbourne, Mt Carty Ridge, and Hercules Nevè, with concentrations ranging from 70 to 1260 ng L^−1^ [201]. To the best of our knowledge, phthalate contamination in snow at high altitudes has been detected so far only at the Sonnblick Observatory on Mt. Sonnblick in the Austrian Alps. In these samples concentrations ranged up to 40,000 ng L^−1^, resulting among the dominant lipidic compounds found in snow [202].

**Bisphenol A (BPA),** a monomer initially synthesised as a synthetic oestrogen in the 1890s [208], was later incorporated into a wide range of consumer products, including plastics, PVC, food packaging, epoxy resins, dental sealants, and thermal receipts [209]. Today, its endocrine-disrupting effects are well-established [210,211], but its widespread use and irregular processing methods have led to BPA being detected globally [212]. Its presence in snow was first documented in Germany [213] and Minnesota [214]. However, the first quantification of BPA in Arctic snow was reported by [18], who found it at concentrations ranging between 0.2 ng L^−1^ and 65 ng L^−1^. In Antarctica, the presence of BPA was detected in sea ice at concentrations ranging from <1.3–3.8 ng L^−1^ [203]. Conversely, to the best of our knowledge, the occurrence of BPA in snow and ice from high-altitude glaciers has not yet been studied, likely due to limitations in routinary instrumentation, as well as high water solubility and low persistence of these compounds that can degrade due to the exposure to UV radiations [215].

### 4.3. POPs-EDCs

POPs-EDCs compounds display intermediate behaviour: on one hand, they may be characterised by prolonged persistence, bioaccumulation potential, and LRT mobility [216,217]. On the other hand, they present endocrine disruptive potential [218]. This includes specific polychlorinated biphenyls (PCBs), polybrominated diphenyl ethers (PBDEs), PFAS, organochlorine pesticides (OCPs) and organophosphate esters (OPEs) (Table 4) [219].

**Polychlorinated biphenyls (PCBs)** are a mixture of up to 209 chlorinated congeners ranging from oily liquids to waxy solids [220]. Considered key Arctic indicator contaminants for trend and risk assessment [221,222,223], PCBs have shown declining environmental concentrations over the past decades, largely as a consequence of reduced emissions following national bans introduced in the 1970s–1980s and the global restrictions enforced under the Stockholm Convention in the early 2000s [222]. Nevertheless, PCBs continue to be deposited in remote cryospheric environments, where systematic monitoring provides direct evidence of their presence. For instance, at Svalbard, the 2014 annual surface snow was collected from Holtedahlfonna and Kongsvegen (western sites), Austfonna (eastern site), and Lomonosovfonna (central Svalbard), and analysed for 209 PCBs congeners. Results revealed the highest ∑PCB flux at Holtedahlfonna and Kongsvegen (26.7 pg cm^−2^ yr^−1^, Kongsvegen), while the lowest fluxes were found at Lomonosovfonna (14.4 pg cm^−2^ yr^−1^), in central Svalbard [13]. The greatest difference between sites was due to the trichlorobiphenyl homologue, which was nearly four times greater at Kongsvegen than the eastern site at Austfonna. Air mass back trajectories from likely source areas for all sites were similar, indicating no difference in frequency or distribution of PCB from long distances, suggesting local PCB sources contributing to Kongsvegen. Despite geographical isolation and almost complete absence of human settlements, traces of PCBs were recorded also in surface Antarctic snow, which recorded a 200% increase in a very limited period (1930–1980). This increase in PCB concentrations was almost totally attributed to the massive industrial production [224]. Moreover, Antarctic snow was sampled from five selected locations of Northern Victoria Land during the austral summer 2011–2012, where the total concentrations of these pollutants (ΣPCBs) were quantified between 0.11 and 0.58 ng L^−1^, showing a net decrease with respect to the past decades [177]. In addition, highly time-resolved PCB’s record is also available from an alpine ice core drilled at the Fiescherhorn glacier, Switzerland, in 2002, whose record covers the entire period of industrial use of PCBs (1940–2002). The total concentration of the six quantified PCBs varies from 0.5 to 5 ng L^−1^ and reveals a temporal trend with an 8-fold increase from the early 1940s to the peak value in the 1970s. Notably, the level in 2002 is comparable to the concentration in the 1940s, when PCBs were introduced into the market. These fluctuations were explained, in addition to changing emissions, by variability in convective transport and postdepositional processes. Overall, concentrations of PCBs revealed at this site appeared in agreement with data from seasonal snow samples in the Alps but revealed a factor of 100 times higher than concentrations measured in the Arctic [225].

**Polybrominated diphenyl ethers (PBDEs)** are a class of organobromine compounds used as flame retardants in a variety of consumer products, like electrical equipment, construction materials, coatings, textiles and polyurethane foam [226]. PBDEs are structurally similar to PCBs, thus have similar properties and the same large number of congeners (i.e., 209) depending on the number and positions of the bromine atoms on the two phenyls rings [227]. Persistence, bioaccumulation, high toxicity and LRT are also characteristics of PBDEs. In the Arctic cryosphere, 15 PBDE congeners were inspected in the upper 34 m of an ice core from Holtedahlfonna (representing 1953–2005), the westernmost ice sheet on Svalbard, finding BDE-209 as dominant congener with quasi-continuum profiles in the ice core and a flux peak of 322 pg cm^−2^ yr^−1^ in the surface layer (1995–2005) [228]. Other PBDEs with long records in the core included BDEs 71 and 109, with peak inputs <1 pg cm^−2^ yr^−1^. High decaBDE fluxes ranging from 30 to 4000 pg cm^−2^ yr^−1^ were measured in snow samples dated 1995–2008 from Devon ice cap, in the central Canadian Arctic Archipelago [229]. In Antarctica, snowmelt concentrations of PBDEs in continental (East Antarctic Plateau) and coastal (Northern Victoria Land) surface snow ranged from 0.13 ng L^−1^ to 0.34 ng L^−1^, with BDE-47 and BDE-99 contributing the most [177]. These concentration ranges and composition profiles agreed well with other observations of [230] in the Western Antarctic Peninsula. Evidence of PBDEs in snow and firn samples have also been reported for mid-latitude high-altitude glaciers. Eight PBDEs congeners were indeed measured in a 10 m shallow firn core from Colle Gnifetti, in the Monte Rosa massif, with BDE 99 and BDE 47 found at concentrations up to 4.5 ng L^−1^ and 2.6 ng L^−1^, respectively [231]. Similarly, PBDEs quantification from a shallow firn core retrieved at Alto dell’Ortles, in the Italian Eastern Alps, revealed the highest concentrations for BDE 47, 99, and 209, with values ranging from <LOQ–8.1 ng L^−1^, 0.1–21.7 ng L^−1^, and 0.1–12.9 ng L^−1^ [232]. Moreover, in the Tyrolean Alps, PBDEs were found in snow samples at concentrations ranging from 0.008 to 0.3 ng L^−1^, with predominance of BDE-47, 99, 100 and BDE-209 among the fourteen investigated BDE congeners [233]. These results were in line with concentrations found in the Tatra Mountains (Slovakia) [234], confirming the capacity of these low-volatile compounds for LRT from distance sources.

**Per- and poly-fluoroalkyl substances (PFAS)** are a diverse group of organofluorine contaminants used in industrial and commercial applications since the 1950s [235]. While regulations under the Stockholm Convention have restricted many PFAS [236], short-chain compounds (e.g., PFHxA and PFBS) and polymeric variants remain unlisted, making them object of growing interest and crucial compounds to investigate in the cryosphere [14]. Their ubiquity extends indeed to remote environments, where snow pits and ice cores record long-term atmospheric deposition [237,238], while surface snow reflects local precipitation events [170].

In high-Arctic ice cores, PFOA was detected at ∼0.06 ng L^−1^ during 1977–2015, representing background atmospheric deposition [238,239,240]. In 2019, surface snow from Svalbard, 36 PFAS, including perfluorobutane sulfonamide (FBSA), perfluorohexane sulfonamide (FHxSA), perfluorooctane sulfonamide (FOSA), N-methyl perfluorobutane sulfonamide (MeFBSA), and N-methyl perfluorooctane sulfonamidoethanol (MeFOSE), were detected at high concentrations, reflecting LRT of precursors to persistent perfluorobutane sulfonate (PFBS), perfluorohexane sulfonate (PFHxS) and perfluorooctane sulfonate (PFOS). Emergent PFAS such as fluoroteromer sulfonate acids (4:2 FTSA, 6:2 FTSA, 8:2 FTSA), fluoroteromer unsaturated carboxylic acids (6:2 FTUCA, 8:2 FTUCA), perfluoroethylcyclohexane sulfonate (PFECHS), chlorinated perfluoroethyl sulfonate (6:2 Cl-PFESA, 8:2 Cl-PFESA), hexafluoropropylene oxide dimer acid (HFPO-DA), and 4,8-Dioxa-3H-perfluorononanoic acid (ADONA) were quantified in ice from Drønbreen, Lomonosovfonna, and Nordmannsfonna glaciers, with concentrations ranging from 0.003 ng L^−1^ (HFPO-DA) to 0.077 ng L^−1^ (6:2 FTUCA) [14].

In Antarctica, 16 PFAS were detected in snow from the Dome C ice core (Dome Concordia, Wilkes Land, East Antarctica) in 2016, with dominant PFOA, followed by perfluoroheptanoic acid (PFHpA), perfluorohexanoic acid (PFHxA), and perfluoropentanoic acid (PFPeA) [239]. Long-chain PFCA (C9-C14) represented ~10% of total PFCAs, while PFOS, perfluorobutane sulfonate (PFBS), and PFHxS were the main PFSAs. HFPO-DA was detected for the first time in Antarctic snow at 0.005–0.013 ng L^−1^ [239,241].

High-altitude glaciers also recorded PFAS contamination. A firn core from Mt Ortles, Italy, contained 12 PFAS, with ΣPFAS ranging 1 ng L^−1^–5.8 ng L^−1^, and PFOA, PFBA, PFNA present at low ng L^−1^ concentrations [232]. Moreover, in Klippitztorl, Austria, 16 PFAS were identified in snowmelt with ΣPFAS up to 143 ng L^−1^, mainly linked to ski wax use [33]. On Mt Oxford, Ellesmere Island, Canada, PFCA reached 31.6 pg cm^−2^ area^−1^ and PFOS/PFBS were elevated up to 47.1 pg cm^−2^ area^−1^ in pre-1990s samples [242].

**Organochlorine pesticides (OCPs)** are known for their slow degradation, high toxicity, bioaccumulation potential and long-range mobility [161]. Although many OCPs have been banned in developed countries, their use persists and is even increasing in developing regions, where they remain employed for economically driven agricultural practices and, in some cases, for public-health vector control (e.g., Dichlorodiphenyltrichloroethane—DDT—for malaria) [243]. Their continued application is further sustained by weak regulatory oversight and limited capacity for monitoring and managing obsolete pesticide stocks [244]. Owing to this persistence and high environmental mobility, OCPs can reach remote regions via long-range atmospheric transport, as testified by the 36 classes detected in winter snow from four glacial sites on Svalbard, at varying elevations (i.e., Lomonosovfonna, Austfonna, Holtedahlfonna, and Kongsvegen) with dominating contribution of trans-chlordane, dieldrin, and chlorpyrifos that together comprised at least 50% of total OCP at each site [13]. ∑OCPs fluxes at Lomonosovfonna, Austfonna, Holtedahlfonna and Kongsvegen were measured as 190.5, 77.3, 103.5 and 173.5 pg cm^−2^ yr^−1^, respectively (Table 4). In Antarctica (Dome Summit South and Law Dome), OCPs, including hexachlorocyclohexanes (HCHs with its isomers: α-HCH, β-HCH, γ-HCH, δ-HCH), heptachlor (HEPT), trans-chlordane (TC), dieldrin (DIE) and endrin (END), were first quantified in archived firn cores representing the 1945–1957 and 1958–1967 periods, with firn deposition rates found below 0.8 pg cm^−2^ yr^−1^ for α and γ-HCH, heptachlor, trans-chlordane and endrin, and dieldrin deposition rates of up to 4 pg cm^−2^ yr^−1^ [245]. OCPs were also measured in surface snow collected on a 1400 km inland traverse beginning from the coastal regions of East Antarctica during the Japanese Antarctic Research Expedition (JARE) of 2007/2008, revealing concentration ranges of 0.02–0.08, 0.03–0.14, and 0.18 ng L^−1^ for α-HCH, γ-HCH, and hexachlorobenzene (HCB), respectively [246]. Among the 22 OCPs identified, the most abundant was γ-HCH, with a mean concentration of 0.07 ng L^−1^, followed by α-HCH, with an average concentration of 0.04 ng L^−1^. The spatial variability of α-HCH and γ-HCH was narrow, and the concentrations of α-HCH and γ-HCH increased slightly with increasing altitude along the traverse route. Dome Fuji, the highest altitude sampling point, had the highest γ-HCH concentrations in the snow. Backward air trajectory analysis showed that the air masses at the sampling sites came mainly from the Indian and Atlantic Oceans and over the Antarctic continent, indicating that the OCPs were subjected to long-range atmospheric transport and were deposited in the surface snow. Furthermore, OCPs were quantified in snow sampled in the Western Antarctic Peninsula [230]. In this case, the sum of DDT’s six isomers showed the highest concentrations among all the investigated OCPs at all the locations (Old Palmer, Palmer Backyard, Palmer Glacier, Torgerson’s Island, Jacob’s Island), with concentrations ranging from 0.03 ng L^−1^ (Jacob’s Island) to 0.4 ng L^−1^ (glacier). Aldrin, dieldrin, oxychlordane, endosulfans, endrin, endrin aldehyde, endrin ketone and methoxychlor were instead below LOD in all samples. OCPs (i.e., α-HCH, γ-HCH, HCB, and DDT) were also quantified in surface snow and ice cores from high-altitude glaciers since the early 2000s (e.g., [247,248]). Notably, Carrera et al. (2001) [248] reported concentrations in snow for Tatra, Caledonian mountains, Alps, Pyrenees of PCBs: 0.2–2.2, HCH: 0.02–1.1, DDTs: 73–130, PAHs: 5.6–81 ng L^−1^, with possible 10 times underestimation due to volatilisation from snow. Villa et al. (2003) [247], on the contrary, measured substantially higher deposition fluxes on the Lys glacier in comparison with those calculated for the European mountains at lower altitude, and, in general, closer to deposition values estimated from lake sediment sampled in the European mountains and in the Arctic. For snow sampled in 2000, for instance, deposition fluxes reported for α-HCH, γ-HCH, HCB, and DDT were 310, 186, 21.7, and 37.2 pg cm^−2^ yr^−1^, respectively. Ice cores and meltwater samples from the polythermal Jarvis glacier (Alaska) were analysed for DDT, DDE, DDD, α-HCH, and γ-HCH [174], showing the highest concentrations for DDT (0.51 ng L^−1^), followed by DDD (0.45 ng L^−1^) and DDE (0.34 ng L^−1^). Both α-HCH, and γ-HCH were found at much lower concentrations (0.15 ng L^−1^) throughout all layers. Generally, concentrations found within the ice core were lower than concentration found in the meltwater, potentially a result of increasing chemical loss from the ablating, polythermal glacier.

**Organophosphate esters (OPEs)**, classified into chlorinated and non-chlorinated types based on their substituents, have recently gained extensive attention as synthetic industrial chemicals widely used as flame retardants, plasticisers, and additives in industries such as electronics, construction materials, and consumer goods [249,250]. The total average concentration of OPEs (∑OPEs) in surface Antarctic snow ranges from 0.70 ng L^−1^ in East Antarctica [251] to 12.61 ng L^−1^ at Dome C [167], whereas average concentrations of 5.84 ng L^−1^ were recorded in Arctic snow [252].

**Table 4:** Summary of POPs-EDCs, including geographic areas, sampling sites, target compounds, matrices, concentration ranges (ng L^−1^), fluxes (pg cm^−2^ yr^−1^), and corresponding references.

Category of ECs	Classes	Geographic Area	Sites	Species	Matrix	Concentrations (ng L^−1^)	Fluxes (pg cm^−2^ yr^−1^)	References
POPs-EDCs	Polychlorinated biphenyls (PCBs)	Svalbard	Kongsvegen, Lomonosovfonna, Austfonna, Holtedahlfonna	trichlorobiphenyl	snow		∑PCB: 26.7, Kongsvegen; 14.4 Lomonosovfonna	[13]
		Antarctica	Northern Victoria Land	PCBs: PCB-11, I-PCB-28, I-PCB-52, I-PCB-101, I & DL-PCB 118, I-PCB 138, I-PCB-153, I-PCB-180, DL-PCB- 105, DL-PCB-156, DL-PCB-167, DL-PCB-189, MoCBs, DiCBs, TriCBs, TeCBs, PeCBs, HxCBs, HpCBs, OcCBs, ∑127 PCB, ∑7 PCBs	snow	ΣPCBs: 0.11 and 0.58		[177]
		Alps	Fieschernhorn Glacier	PCB 28, PCB 52, PCB 101, PCB 138, PCB 152, PCB 180	ice	from 0.5 to 5		[225]
	Polybrominated diphenyl ethers (PBDEs)	Arctic	Holtedahlfonna	BDE-209, BDE-71, BDE-109	ice		BDE-209: 322, BDE-71 and BDE-109: <1	[228]
		Canadian Arctic	Devon ice cap	decaBDE	snow		fluxes ranging from 30 to 4000	[229]
		Antarctica	Northern Victoria Land	BDE-47, BDE-99	snowmelt	from 0.13 to 0.34		[177]
		Alps	Colle Gnifetti	BDE-47, BDE-99	firn	BDE-47: up to 2.6, BDE-99: up to 4.5		[231]
		Alps	Ortles Glacier	BDE-47, BDE-99, BDE-209	firn	BDE-47: from <LOQ–8.1, BDE-99: 0.1–21.7, BDE-209: 0.1–12.9		[232]
		Alps	Tyrol	BDE-47, BDE-99, BDE-100, BDE-209	snow	from 0.008 to 0.3		[233]
	Per-poly-fluoroalkyl substances (PFAS)	Svalbard	Longyearbyen, Kjell Henriksen Observatory, Foxfonna ice cap	Emergent PFAS: fluoroteromer sulfonate acids (4:2 FTSA, 6:2 FTSA, 8:2 FTSA), fluoroteromer unsaturated carboxylic acids (6:2 FTUCA, 8:2 FTUCA), perfluoroethylcyclohexane sulfonate (PFECHS), chlorinated perfluoroethyl sulfonate (6:2 Cl-PFESA, 8:2 Cl-PFESA), hexafluoropropylene oxide dimer acid (HFPO-DA), and 4,8-Dioxa-3H-perfluorononanoic acid (ADONA)	snow and ice	from 0.003 (HFPO-DA) to 0.077 (6:2 FTUCA)		[14]
		Alps	Ortles Glacier	ΣPFAS	firn	ΣPFAS ranging 1–5.8		[232]
		Alps	Klippitztorl Glacier	ΣPFAS	snowmelt	up to 143		[33]
		Canadian Arctic	Mt Oxford, Ellesmere Island	PFCA, PFOA, PFNA	ice		PFCA reached 31.6, PFOA and PFNA were found at concentrations below 0.2, and PFOS/PFBS were elevated up to 47.1	[242]
	Organochlorine pesticides (OCPs)	Svalbard	Kongsvegen, Lomonosovfonna, Austfonna, Holtedahlfonna	Heptachlor, Heptachlor Epoxide B, Aldrin, α-HCH, γ-HCH, chlorpyrifos, trans-Chlordane, cis-Chlordane, 4,4′-DDE, Dieldrin, Dachtal, trans-Nonachlor, Endosulfan I	snow		∑OCP Lomonosovfonna: 190.5, Austfonna: 77.3, Holtedahlfonna: 103.5, Kongsvegen: 173.6	[13]
		Antarctica	Dome Summit South, Law Dome	hexachlorocyclohexanes (HCHs with its isomers: α-HCH, β-HCH, γ-HCH, δ-HCH), heptachlor (HEPT), trans-chlordane (TC), dieldrin (DIE) and endrin (END)	firn		0.8 for α and γ-HCH, heptachlor, trans-chlordane and endrin, and dieldrin deposition rates of up to 4	[245]
		Antarctica	Syowa Station, Dome Fuji, turning point (1400 km traverse)	γ-HCH, α-HCH	snow	α-HCH: 0.02–0.08, γ-HCH: 0.03–0.14, hexachlorobenzene (HCB): 0.18		[246]
		Antarctica	Western Antarctic Peninsula: Palmer Station, Jacob’s Island, Torgerson	DDTs six isomers	snow	from 0.03 (Jacob’s Island) to 0.4 (glacier). Aldrin, dieldrin, oxychlordane, endosulfans, endrin, endrin aldehyde, endrin ketone and methoxychlor were instead below LOD in all samples		[230]
		Tatra, Caledonian mountains, Alps, Pyrenees	Starolesnianske (Tatra), Redó lake (Pyrenees), Gossenkölle lake (Alps), Jöri lake (Alps), Øvre Neådalsvatn lake (Caledonian mountains)	DDTs, HCBs, HCH	snow	HCH: 0.02–1.1, DDTs: 73–130		[248]
		Alps	Col del Lys	α-HCH, γ-HCH, HCB, DDT	snow and ice		α-HCH: 310, γ-HCH: 186, HCB: 21.7, DDT: 37.2	[247]
		Alaska	Jarvis glacier	DDT, DDE, DDD, α-HCH, γ-HCH	ice and meltwater	DDT: 0.51, DDD: 0.45, DDE: 0.34, α-HCH: 0.15, γ-HCH: 0.15		[174]
	Organophosphate esters (OPEs)	Antarctica	Dome C	∑OPEs	snow	from 0.70 in East Antarctica to 12.61 at Dome C		[167]
		Arctic	Arctic sea ice	∑OPEs	snow	5.84		[252]

### 4.4. POPs-like

The POPs-like class are a group of ECs characterised by high persistence in the environment, high toxicity, bioaccumulation potential and global distribution. However, they are not formally listed as POPs under the Stockholm Convention because they are largely unintentional by-products [253].

This class includes **Polycyclic Aromatic Hydrocarbons (PAHs)**, which are compounds with two or more benzene rings. These substances are primarily originated from incomplete combustion of organic matter and fossil fuels, which makes them valuable tracers of combustion processes [254]. Major anthropogenic sources of PAHs include residential heating, coal processing, carbon black, asphalt, and aluminium production, although natural sources such as open burning, petroleum or coal seepage, and volcanic activity also contribute [255]. PAHs are ubiquitous in various environmental matrices (e.g., [172]) across both populated and remote regions [256], including snow and ice across high-latitudes and high-altitudes (Table 5). Notably, PAHs have been found in glaciological records from Greenland with ∑PAHs ranging 0.1–10.6 ng L^−1^ [257], and from 0.6 to 2.4 ng L^−1^ [258], melting glacier snowpack in Svalbard with ∑PAHs concentrations from 0.86 to 37 ng L^−1^ [259,260], Antarctica with ∑PAHs ranging from 1.6 to 4.4 ng L^−1^ [167], Himalayan region with ∑PAHs below 100 ng L^−1^ [261], and the Alps with 32 ng L^−1^ [256] (Table 5). Although their presence in the snow, firn, and ice of Arctic glaciers has been studied since the mid-1990s [262], only recent work by Ju et al. (2025) [263] examined PAHs partitioning from melting glaciers into frozen lakes, finding that over 87% of low-ring PAHs remain trapped in the ice column as aerosols, whereas more than 88% of total PAHs are bound to SPM in the water column. Their distribution is strongly controlled by their physiochemical properties, natural conditions, and anthropogenic inputs, highlighting the need for revised monitoring protocols to address SPM-mediated PAHs transport. Although many PAHs are now regulated, i.e., the EU has recently strengthened REACH restrictions on 18 PAHs [264], significant challenges remain, particularly regarding environmental monitoring, LRT, and the management of PAHs released from melting ice and particulate matter.

## 5. Pharmaceutical Compounds and Personal Care Products (PPCPs)

Pharmaceutical contaminants (PCs) and personal care products (PCPs), generally referenced as PPCPs, are among the major groups of emerging contaminants which can cause adverse effects on living organisms even at very low concentrations (ng L^−1^ to µg L^−1^) [265]. These pollutants, whose typical sources are sewage effluents, hospital and animal waste, are characterised by persistence, bioaccumulation potential, and toxicity [266]. Specifically, PCs are a wide variety of biological compounds used for the treatment of infections and diseases, classified into different classes based on their mechanism of action, chemical structures, and treatment scope. The major classes are analgesics and anti-inflammatories (e.g., aspirin, diclofenac, ibuprofen, paracetamol, acetylsalicylic acid, and naproxen), antibiotics (e.g., tetracyclines, penicillins, sulfamethoxazole, erythromycin, trimethoprim) [265], antidepressant (e.g., benzodiazepines, fluoxetine), antiepileptics (e.g., carbamazepine), β-blockers (e.g., atenolol, propranolol, and metoprolol), and antihistamines (e.g., ranitidine and famotidine) [267]. Other substances include illicit drugs like cocaine, barbiturates, methadone, amphetamines, opiates, heroin, and other narcotics [268]. PCPs are a class of emerging pollutants which include instead sunscreens, body lotions, shampoos, toothpaste, detergents, hair dyes, cosmetics, fragrances, and other household items [269].

Over the last few decades, there has been a massive hike in the manufacturing and use of PPCPs, which has been reflected into a sharp increase in these bioactive chemicals in water bodies (e.g., [265]). Although PPCPs have been presented in water for decades, their levels in the environment have only recently begun to be quantified thanks to the advancements in analytical techniques and acknowledged as potentially hazardous to ecosystems [267,270]. Notably, in 41 countries it has been reported the presence of 203 pharmaceutical drugs disposed in river water [271], which led to growing concern, as these contaminants are easily passed through the drinking water and irrigation water resources, reaching the biological system [272]. However, PPCPs have not been considered as priority pollutants in the cryosphere until recent years, as most of them were expected to be found close to their primary sources [273]. Recent studies showed that the decentralised character of local PPCPs sources, in combination with low temperatures and low technological standards for waste treatment facilities in remote environments, considerably extend the environmental stability of these residues compared to most populated regions [273,274]. Therefore, pilot studies on PPCPs started to focus on snow and ice matrices sampled in remote environments (Table 6).

In Arctic, PPCPs levels were first determined in effluent water samples and receiving sea water from three typical waste water treatment plants (WWTPs) in Norway, from Oslo (60° N) Tromsø (70° N) and Longyearbyen, Svalbard (78° N) [275]. Notably, traces of anti-inflammatories (i.e., ibuprofen, diclofenac), antibiotics (i.e., triclosan, tetracycline, trimethoprim, sulfamethoxazole) and antidepressants (i.e., citalopram, fluoxetine, norfluoxetine, fluvoxamine, sertraline, desmethylsertraline, and paroxetine) were found [273]. However, to the best of our knowledge, only Spataro et al. (2025) [19] have quantified the presence of pharmaceuticals in snow and ice. In particular, antibiotics (i.e., ciprofloxacin, enrofloxacin, amoxicillin, erythromycin, sulfamethoxazole, and N4-acetylsulfamethoxazole), anti-inflammatories (i.e., diclofenac, ibuprofen, acetylsalicylic acid, paracetamol), anticonvulsants (carbamazepine), and hormones (i.e., 17α-ethinyl estradiol, 17β-estradiol) were detected in samples collected during the 2022 and 2023 campaigns in Svalbard. However, among the 13 targeted PCs, only enrofloxacin and paracetamol were consistently detected in snow and ice core samples, at concentrations ranging from <1 ng L^−1^ to 17 ng L^−1^ for paracetamol and from 4 ng L^−1^ to 15 ng L^−1^ for enrofloxacin. Within the same study, triclosan (TCL), N-diethyl-meta-toluamide (DEET), and estrone (E1) were investigated as PCPs in snow and ice, revealing the consistent presence of only DEET and E1 in snow and ice core samples, with mean concentrations of 20 ng L^−1^ and 30 ng L^−1^ for DEET and E1, respectively. PCPs were also investigated in the snowpack on north-western Spitzbergen by D’Amico et al. (2024) [18]. Overall, 7 fragrances (FMs) were inspected: amyl and isoamyl salicylate (IsoamylS), hexyl salicylate (HexS), benzyl salicylate (BenS), oranger crystals (OraC), peonile (Peo), and ambrofix (Amb). In addition, organic UV filters (UVFs) were quantified: 2-ethylhexyl 4-methoxycinnamate (EHMC), benzophenone-3 (BP3), ethylhexyl salicylate (EHS), and octorylene (OCR). Results shown that the total FM concentrations range from 1.4 ng L^−1^ to 148 ng L^−1^, with the highest concentration detected for HexS (56 ng L^−1^). The concentrations found in this study were similar to those found in snow samples in the area around Ny-Ålesund by Vecchiato et al. (2018) [260], up to 72 ng L^−1^, and to the ones found in Antarctic seawater during the melting season [276] (intermediate range of 18-64 ng L^−1^). Conversely, these concentrations were lower than the maximum concentrations found in an ice core from Mt Elbrus (Caucasus) by Vecchiato et al. (2020) [277]. The concentration of the 4 UVFs ranged instead from 0.2 ng L^−1^ to 358.4 ng L^−1^, with dominant UVFs represented by EHS and BP3, with concentrations that account for 64% and 18% of the total concentrations of the UVFs in the 25 snow samples, respectively. To the best of our knowledge, no studies to date have reported concentrations of PPCPs in snow and ice from high altitude glaciers, revealing a significative gap-in-the-knowledge.

**Table 6:** Summary of two classes of PPCPs, including geographic areas, sampling sites, target compounds, matrices, concentration ranges (ng L^−1^), fluxes (pg cm^−2^ yr^−1^), and corresponding references.

Category of ECs	Classes	Geographic Area	Sites	Species	Matrix	Concentrations (ng L^−1^)	References
Pharmaceutical compounds and personal care products (PPCPs)	Pharmaceutical Compounds (PCs)	Svalbard	Austre Brøggerbreen, Midre Lovenbree, Kongsvegen	paracetamol, enrofloxacin, N-diethyl-meta-toluamide (DEET), estrone (E1)	snow and ice	paracetamol: <1 to 17, enrofloxacin: from 4 to 15, DEET: 20, E1: 30	[19]
	Personal Care Products (PCPs)	Svalbard	Edithbreen, Midtre Lovénbreen, Austre Brøggerbreen, Kongsvegen, Holtedahlfonna	Seven fragrances: amyl and isoamyl salicylate (IsoamylS), hexyl salicylate (HexS), benzyl salicylate (BenS), oranger crystals (OraC), peonile (Peo), and ambrofix (Amb); 4 UV filters (UVFs)—with dominant EHS and BP3	snow	total FM from 1.4 to 148. Highest concentration detected for HexS: 56. UVFs: from 0.2 to 358.4	[18]
		Svalbard	transect along the Brøgger peninsula	Bourgeonal, Amyl Salicylate, Oranger Crystals, Hexyl Salicylate, Ambrofix, Peonile, Benzyl Salicylate	snow	up to 72	[260]
		Caucasus	Mt Elbrus	Amyl Salicylate, Oranger Crystals, Hexyl Salicylate, Ambrofix, Peonile, Okoumal, Benzyl Salicylate	ice	peak in 2004: 281	[277]

## 6. Emerging Contaminants and Climate Change: Ecotoxicological Risks for Wildlife and Human Health in Polar and High-Mountain Regions

Ecosystems monitoring and health risk assessment of ECs in polar and high-mountain regions have emerged as significant research priorities [11]. Indeed, unregulated hazardous pollutants are released at an alarming rate from glaciers due to the acceleration of ice and snow melt driven by climate change [9]. Furthermore, ECs and climate change have been demonstrated to interact with numerous other environmental stressors, including habitat destruction and biodiversity, increasing human migration, resource exploration and extraction, and tourism activities, resulting in both direct and indirect responses in ecosystems and organisms [274,278].

For example, in the Arctic, which is among the fastest-warming regions due to climate change (e.g., [279]), endocrine-disrupting effects of synthetic chemicals appear to be amplified by the gradual disappearance of sea ice [15,280]. Resulting effects are monitored in ice-associated sentinel species, like polar bears and narwhals (*Monodon monoceros*), which seem to experience a reduction in their ability to reproduce and undergo normal grow and development due to these combined and magnifying effects [281]. Temperature increases have been shown to influence the LRT of POPs to the Arctic and their re-mobilisation from secondary sources [16]. These processes may contribute to increased exposure of Arctic biota to POPs. The Arctic biota has shown elevated POPs concentrations associated with climate oscillation indices, precipitation, water salinity and sea ice age variations modulated by the ongoing climate change [278]. Biota has demonstrated to act as a biovector for POPs and contaminants of emerging Arctic concern (CEACs) into Arctic marine food webs. These exposure pathways have been associated with immune alterations in top predators, although the magnitude of population-level effects remains to be fully clarified [280]. The accumulation of CEACs in addition to legacy POPs increases mixture complexity. Experimental and field evidence suggest that such mixtures may amplify cumulative and synergistic effects on exposed wildlife, potentially increasing the likelihood of immune, reproductive, and neuroendocrine thresholds being exceeded [282]. In addition, many chemical degradation products that can form either in the atmosphere during LRT to the Arctic or can be biotransformed in animals, like 1,1-dichloro-2,2-bis(4-chlorophenyl)ethene (p,p′-DDE), require increasing attention, as they can have potential harmful biological effects [280].

While the effects of climate on POP trends in the Arctic have garnered attention, studies on these effects in the Antarctic have been limited until recently (e.g., [283]). Recent findings show that climate-related stressors are also reducing the resilience of Antarctic organisms, heightening the risks of emerging contaminants, especially in coastal areas influenced by human activities [10]. Research at McMurdo Station in coastal benthic communities highlights the role of sea ice in accumulating persistent contaminants from atmospheric deposition and seawater, which may be taken up by sea ice algae and other organisms in cryopelagic communities [284]. Additionally, seawater acidification, a consequence of climate change, is emerging as a stressor that may affect the resilience of Antarctic marine organisms, making them more sensitive to emerging contaminants.

Outside the poles, the Third Pole region, which includes the Tibetan Plateau and surrounding alpine ecosystems, is highly sensitive to global warming. As climate change accelerates glacial melting and permafrost thaw, contaminants are released into downstream environments, raising concern about their long-term impacts on water quality, aquatic life, and human health [9]. However, significant knowledge gaps remain regarding the quantitative fluxes of these releases and their transformation pathways, requiring a comprehensive risk assessment framework to quantify the extent of contamination and evaluate potential health effects [285]. The release of Re-MMs like Hg and Cd, and of CCH-MMs such as Pb and As from glaciers into freshwater systems has been documented. Given their toxicity, persistence, and bioaccumulation potential, these inputs may represent ecological and human health risks, particularly when concentrations exceed the WHO drinking water guidelines [9]. Metal and metalloid exposure has been associated with a range of adverse health problems in humans, including neurological disorders, kidney damage, and cardiovascular illness (e.g., [286]). Particularly, the consumption of mercury-contaminated fish can lead to neurological disorders, cognitive impairment, and developmental issues, especially in vulnerable populations such as pregnant women and children [287]. Similarly, exposure to POPs like pesticides, industrial chemicals and combustion by-products has been linked to endocrine disruption, reproductive disorders, immune system suppression, and an increased risk of cancer in human populations [9]. Recent studies have documented significant concentrations of DDT and PCBs in meltwater from glaciers in the European Alps [31] and the Canadian Rockies [288], indicating the global scale of contamination, present in both water and biota and transferable through the food web [289]. Populations in high-altitude and downstream regions are particularly vulnerable to contamination released from glaciers, which can infiltrate groundwater systems, making remediation efforts challenging due to the persistence and mobility of pollutants. In addition, the increasing frequency of glacial lake outburst floods due to climate change poses an additional hazard, as these events can rapidly transport large volumes of contaminated sediments into downstream ecosystems, which may contribute to elevated pollutant transport [290].

These findings document increasing contaminant mobilisation under ongoing cryospheric change and support the need for systematic monitoring efforts. Models can assist in exploring how contrasting climate-driven processes may influence emerging pollutant exposures and in estimating potential human exposure via drinking water, dietary intake, and recreational activities [291]. However, insufficient data exists with which populate such models, especially for food web dynamics [278]. Furthermore, the dynamic characteristics of glaciers and meltwater streams generate temporal fluctuations in pollutant concentrations, underscoring the importance of long-term observations to better distinguish between measured trends and projected risk scenarios [9].

## 7. Current Regulatory Frameworks

Polar regions are increasingly threatened by the presence of ECs [292]. Owing to their extensive use and environmental persistence, many of these chemicals remain in the environment for decades and are redistributed globally via successive volatilisation and condensation cycles [9]. Although hundreds of ECs have been detected in the cryosphere, only about 35 chemical groups are currently listed or proposed for global regulation under the Stockholm Convention, despite an estimated 350,000 chemicals in use worldwide and the continuous introduction of new compounds [280]. The implementation of the original 12 POP classes (“Dirty Dozen”) has contributed to declining concentrations in Arctic biota [293], yet most ECs, ranging from PCs to ICBs, remain outside globally harmonised regulatory control. Regulation of ECs is hindered by fragmented legislation, divergent national priorities, and limited data on toxicity, persistence, and environmental behaviour [1,294]. Harmonised monitoring efforts and shared prioritisation strategies are therefore needed, requiring coordinated scientific and policy input at the international level, and investment in monitoring infrastructure [9,295].

Existing policy instruments span precautionary regulations, polluter-pay principles, and integrated water resource management frameworks [296]. In the European Union (EU), REACH and the Water Framework Directive (WFD) represent the main legislative pillars for managing ECs [1]. REACH ensures chemical registration and evaluation, while the WFD sets environmental quality standards and maintains dynamic priority and watch lists [297,298]. The revision of Directive 2020/2184 expanded drinking-water regulation to include pharmaceuticals and hormones. Regional initiatives, such as the EU Water Initiative, support capacity-building and knowledge exchange.

The United Nations and the World Health Organization (WHO) provide guidance for monitoring and managing ECs, offering essential support to countries with limited regulatory infrastructure [299]. In North America, the EPA has taken a leading role in regulating PFAS and other pollutants through the Safe Drinking Water Act, while the Canadian Environmental Protection Act (CEPA) focus on a precautionary approach for emerging pollutants such as pharmaceuticals and industrial chemicals but lags in regulating other ECs like PFAS [294].

Regulatory progress remains uneven elsewhere. Regions like sub-Saharan Africa and Southeast Asian countries rely primarily on the WHO guidelines due to the absence of comprehensive national policies [300]. Australia and Japan enforce extensive monitoring programmes for persistent pollutants and pharmaceuticals, whereas China and India are still developing broader EC frameworks, with existing regulations largely limited to MMs or selected industrial contaminants [294].

## 8. Conclusions and Future Perspectives

A systematic review of metals and metalloids, industrial, pharmaceutical, and personal care emerging contaminants (acronyms summarised in Table 7) in the global cryosphere has been lacking. Although MMs have been widely investigated in polar and alpine environments, they have not yet been clearly categorised within the broader framework of emerging contaminants. This review fills these gaps by offering an integrated global overview of emerging contaminants in snow and ice, and by proposing the first comprehensive classification of metals and metalloids based on recent applications, environmental behaviour, and associated ecological and human-health risks. Three main groups are identified: emerging (Sb, Tl, Ga), re-emerging (Cr, Ni, Cu, Zn, Cd, Hg), and historical MMs of continuous concern (As, Pb). The review also provides a consolidated assessment of industrial emerging contaminants in the cryosphere, distinguishing persistent organic pollutants (POPs) from non-persistent endocrine-disrupting chemicals. Extensive literature is available for several POP categories, particularly OCPs, PFAS, PAHs, and PBDEs, as well as for non-POP EDCs. In contrast, research is limited or nearly absent for POP classes such as PCBs, PCDD/Fs, NBFRs/NCFRs, SCCPs/MCCPs/LCCPs, and OPEs, indicating an urgent need for further investigation in both high-latitude and high-altitude environments. Pharmaceuticals and personal care products remain especially underexplored in the cryosphere, despite evidence that they can cause adverse biological effects even at trace concentrations (ng L^−1^ to µg L^−1^). To date, only a narrow range of pharmaceutical compounds has been examined, only in Svalbard snow and ice, through a pivotal study published in 2025. Given their widespread use, environmental persistence, and demonstrated ability to remain in the environment for decades, their sparse detection in the cryosphere highlights major regulatory gaps. More coordinated and robust action is urgently needed to address the release of these hazardous pollutants from melting ice sheets and glaciers under accelerating climate change.

## Figures and Tables

**Table 1:** Classification of anthropogenic metals and metalloids (MMs) detected in snow and ice, grouped into “Emerging” (E-MMs), “Re-Emerging” (RE-MMs), and “Continued Concern Historical” (CCH-MMs) categories, covering records from the early 20th century to the present day (* indicates mean concentrations averaged over multiple glacial sites in the same region).

“Emerging” Metals and Metalloids (E-MMs)					
E-MMs	Geographic Area	Matrix	Reference Period	Av. Conc. (µg L^−1^)	References
Sb	N. America, Arctic Canada	ice	1842–1996	0.001	[51]
	Alps, Colle Gnifetti	snow	1972–1994	0.05	[52]
	Alps, Dome du Goûter	ice	20th century	0.02	[52]
	Asia, Eastern Tibetan Plateau	snow	2017	0.11 *	[39]
	Asia, Northeastern Tibetan Plateau	snow	2017	0.12 *	[53]
	Asia, Northern Tibetan Plateau	snow	2014	0.09 *	[54]
	Asia, Southern Tibetan Plateau	snow	2015	0.002 *	[55]
	Asia, Western Siberian Lowland	snow	2014	0.04	[56]
	Asia, Mt Everest	firn/ice	2002	0.002	[57]
	Asia, Siberian and Mongolian Altai	ice	2000	0.04	[58]
	Asia, Tien Shan	ice	1953–2004	0.04	[59]
	Antarctica, Dome Fuji	ice	1970–1995	0.0003	[60]
Tl	N. America, Ontario, Canada	snow	2005	0.002	[61]
	Asia, Tien Shan	ice	1953–2004	0.013	[59]
	Asia, Mt Everest	ice	1992–2002	0.0009	[62]
	Greenland, D4	ice	1772–2003	0.0002	[63]
Ga	N. America, Ontario, Canada	snow	2005	0.02	[61]
	Asia, Western Siberian Lowland	snow	2014	0.002	[56]
**“Re-Emerging” Metals and Metalloids (RE-MMs)**					
**RE-MMs**	**Geographic Area**	**Matrix**	**Reference Period**	**Av. Conc. (µg L^−1^)**	**References**
Cr	N. America, Ontario, Canada	snow	2005	0.14	[61]
	S. America, Illimani gl., Bolivia	ice	1919–1999	1.03	[64]
	Alps, Colle Gnifetti	snow	1972–1994	0.35	[52]
	Alps, Dome du Goûter	ice	20th Century	0.14	[52]
	Asia, Eastern Tibetan Plateau	snow	2017	6.4 *	[39]
	Asia, Northeastern Tibetan Plateau	snow	2017	10 *	[53]
	Asia, Mt Everest	ice	1992–2002	0.056	[62]
	Asia, Inylchek	ice	1908–1995	0.36	[65]
	Coast Land, Antarctica	snow	1983–1990	0.001	[66]
Ni	N. America, Ontario, Canada	snow	2005	0.13	[61]
	Alps, Colle Gnifetti	snow	1972–1994	0.22	[52]
	Alps, Dome du Goûter	ice	20th Century	0.14	[52]
	Asia, Eastern Tibetan Plateau	snow	2017	0.9 *	[39]
	Asia, Northeastern Tibetan Plateau	snow	2017	1.6 *	[53]
	Asia, Western Siberian Lowland	snow	2014	0.4	[56]
Cu	N. America, Ontario, Canada	snow	2005	0.4	[61]
	S. America, Bolivia	snow/ice	1988	2.3	[67]
	Alps, Colle Gnifetti	snow	1972–1994	0.4	[52]
	Alps, Dome du Goûter	ice	20th Century	0.14	[52]
	Asia, Northeastern Tibetan Plateau	snow	2017	0.8 *	[53]
	Asia, Northern Tibetan Plateau	snow	2014	0.4 *	[54]
	Asia, Western Siberian Lowland	snow	2014	0.6	[56]
	Asia, Siberian and Mongolian Altai	ice	2000	0.3–0.6	[58]
	Greenland, Summit	snow/ice	1974	0.008	[68]
	Antarctica, Zhongshan Station	ice	1968–2016	0.2	[69]
Zn	N. America, Ontario, Canada	snow	2005	4.7	[61]
	S. America, Bolivia	snow/ice	1988	1.7	[67]
	Alps, Colle Gnifetti	snow	1972–1994	3.2	[52]
	Alps, Dome du Goûter	ice	20th Century	1.8	[52]
	Alps, Mont Blanc	ice	1960–1967	0.03–9.9	[34]
	Asia, Eastern Tibetan Plateau	snow	2017	1.8 *	[39]
	Asia, Northern Tibetan Plateau	snow	2014	8.6 *	[54]
	Asia, Western Siberian Lowland	snow	2014	8.3	[56]
	Asia, Siberian and Mongolian Altai	ice	2000	1.5–3.0	[58]
	Greenland, Dye 3	snow	1983–1984	0.03	[70]
	Antarctica, Zhongshan Station	ice	1968–2016	19.2	[69]
Cd	N. America, Ontario, Canada	snow	2005	0.01	[61]
	S. America, Bolivia	snow/ice	1988	0.064	[67]
	Alps, Colle Gnifetti	snow	1972–1994	0.06	[52]
	Alps, Dome du Goûter	ice	20th Century	0.02	[52]
	Alps, Mont Blanc	ice	1960–1967	0.001–0.13	[34]
	Asia, Northeastern Tibetan Plateau	snow	2017	0.01 *	[53]
	Asia, Northern Tibetan Plateau	snow	2014	0.02 *	[54]
	Asia, Western Siberian Lowland	snow	2014	0.05	[56]
	Asia, Siberian and Mongolian Altai	ice	2000	9.07	[58]
	Asia, Tien Shan	ice	1953–2004	0.027	[59]
	Greenland, Dye 3	snow	1983–1984	0.0007	[70]
	Greenland, D4	ice	1772–2003	0.002	[63]
	Greenland, NEEM	snow	2003–2009	0.001	[71]
	Northwest and central Greenland	snow	2012–2013	0.043	[71]
Hg	North America, Wyoming	ice	1986–1993	0.009	[72]
	Alps, Col du Dôme	snow/ice	1990	0.0004	[37]
	Asia, central Tibetan Plateau	snow	2005–2010	0.001–0.04	[73]
	Greenland, Summit	firn and snow	2001	0.0002–0.002	[74]
**Continued-Concern Historical Metals and Metalloids (CCH-MMs)**					
**CCH-MMs**	**Geographic Area**	**Matrix**	**Reference Period**	**Av. Conc. (µg L^−1^)**	**References**
As	N. America, Ontario, Canada	snow	2005	0.07	[61]
	S. America, Bolivia	snow/ice	1988	3.03	[67]
	Asia, Eastern Tibetan Plateau	snow	2017	0.97 *	[39]
	Asia, Northeastern Tibetan Plateau	snow	2017	1.47 *	[53]
	Asia, Western Siberian Lowland	snow	2014	0.2	[56]
Pb	N. America, Canada, Mt Logan	ice	1981–1998	0.07	[75]
	N. America, Ontario, Canada	snow	2005	0.75	[61]
	S. America, Illimani gl., Bolivia	ice	1919–1999	0.32	[64]
	Alps, Mont Blanc	ice	1960–1967	0.05–7.7	[34]
	Alps, Colle Gnifetti	firn/ice	1900–2000	1–3	[36]
	Asia, Northern Tibetan Plateau	snow	2014	0.09 *	[54]
	Asia, Siberian and Mongolian Altai	ice	2000	2–2.5	[58]
	Asia, Tien Shan	ice	1953–2004	1.6	[59]
	Greenland, Dye 3	snow	1983–1984	0.03	[70]
	Greenland (central)	ice	1990–1998	0.03	[76]
	Greenland, D4	ice	1772–2003	0.06	[63]
	Antarctica, Zhongshan Station	ice	1968–2016	16.26	[69]
	Antarctica, Victoria Land	firn	1978	0.004	[77]
	Antarctica, Coast Land	snow	1983–1990	0.0001–0.009	[78]
	Antarctica, Law Dome	ice	1960–1989	0.0009–0.007	[79]

**Table 2:** Summary of persistent organic pollutants (POPs), including geographic areas, sampling sites, target compounds, matrices, concentration ranges (ng L^−1^) and corresponding references.

Category of ECs	Classes	Geographic Area	Sites	Species	Matrix	Concentrations (ng L^−1^)	References
Persistent Organic Pollutants (POPs)	Dioxins and furans (PCDD/Fs)	Antarctica	Northern Victoria Land	1,2,3,4,6,7,8-heptachlorodibenzo-p-dioxin (HpCDD), octachlorodibenzo-p-dioxin (OCDD)	snow	HpCDD: 0.00013, OCDD: 0.0003–0.0005	[177]
	Chlorinated paraffins (SCCPs, MCCPs, LCCPs)	Sweden	Gothenburg	C10-C13 short-chain chlorinated paraffins (SCCP), C14-C17 medium-chain chlorinated paraffins (MCCP)	urban snow	330 and 32,000	[178]

**Table 3:** Summary of two classes of non-POPs-EDCs, including geographic areas, sampling sites, target compounds, matrices, concentration ranges (ng L^−1^), fluxes (pg cm^−2^ yr^−1^), and corresponding references.

Category of ECs	Classes	Geographic Area	Sites	Species	Matrix	Concentrations (ng L^−1^)	References
Non-POPs—Endocrine Disruptive Chemicals (EDCs)	Phthalates	Russian Arctic	Franz Josef Land Archipelago	dimethyl-, diethyl- and dibutylphthalate	snow	between 10 and 300	[199]
		Russian Arctic	Novaya Zemlya Archipelago	dimethylphthalate	snow	between 20 and 60	[200]
		Antarctica	Wood Bay, Vegetation Island, Mount Melbourne, Mc Carty Ridge, Hercules Nevè	Di-iso-butylphthalate, Di-n-butylphthalate, Bis(2ethylhexyl)phthalate	snow	from 70 to 1260	[201]
		Alps	Mt Sonnblick	Dibutyl-phtalic acid ester, Diisobutyl-phtalic acid ester, Diisooctyl-phthalic acid ester	snow	up to 40,000	[202]
	Bisphenol A (BPA)	Arctic	Edithbreen, Midtre Lovénbreen, Austre Brøggerbreen, Kongsvegen, Holtedahlfonna	4-[2-(4-hydroxyphenyl)propan-2-yl] phenol	snow	between 0.2 and 65	[18]
		Antarctica	Ross Island	BPA	ice	from <1.3–3.8	[203]

**Table 5:** PAHs in the cryosphere. Specifics on geographic areas, sampling sites, target compounds, matrices, concentration ranges (ng L^−1^) and corresponding references.

Category of ECs	Classes	Geographic Area	Sites	Species	Matrix	Concentrations (ng L^−1^)	References
POPs-like	Polycyclic Aromatic Hydro carbons (PAHs)	Greenland	Summit	naphtalene, phenanthrene, anthracene, fluoranthene, pyrene, chrysene, benzo(a)anthracene, benzo(b) and (k) fluoranthenes, benzo(a)pyrene, indenopyrene, benzo(ghi)perylene and coronene and retene.	snow	∑PAHs from 0.1 to 10.63	[257]
		Greenland	Summit	Naphtalene, Phenanthrene, Fluoranthene, Pyrene, Benzoaanthracene, chrysene, benzobfluoranthene, benzokfluoranthene, benzoapyrene, benzoghiperylene, coronene	snow	∑tPAHs from 0.6 to 2.4	[258]
		Svalbard	Ny-Ålesund	Naphtalene, acenaphthylene, acenaphthene, fluorene, phenanthrene, anthracene, fluoranthene, pyrene, benzo(a) anthracene, chrysene, retene, benzo(b)fluoranthene, benzo(k) fluoranthene, benzo(e)pyrene, benzo(a) pyrene, perylene, dibenzo(a,h)anthracene, indeno (1,2,3-c,d)pyrene, benzo(ghi)perylene	snow	∑16 PAHs: between 0.86 and 37	[259]
		Svalbard	transect along the Brøgger peninsula	Naphtalene, acenaphthylene acenaphthene, fluorene, phenanthrene, anthracene, fluoranthene, pyrene, benzo(a)anthracene, chrysene, retene, benzo(b)fluoranthene, benzo(k) fluoranthene, benzo(a)pyrene,	snow	∑16PAHs: between 2.6 and 299	[260]
		Antarctica	Dome C	2-MP, 1-MP, acenaphthylene acenaphthene, fluorene, phenanthrene, anthracene, fluoranthene, pyrene, benzo(a)anthracene, chrysene, benzo(b)fluoranthene, benzo(k) fluoranthene, benzo(a)pyrene, benzo(ghi)perylene, indeno(1,2,3-c,d)pyrene, dibenzo(a,h)anthracene	snow	from 1.6 to 4.4	[167]
		Himalayan region	Rongbuk Glacier, Mt Everest	Naphthalene, Acenaphthene, Acenaphthylene, Anthracene, Fluorene, Fluoranthene, Phenanthrene, Pyrene, Chrysene, Benzo[a]anthracene, Benzo[b] fluoranthene	snow and ice	below 100	[261]
		Alps	Vallebelluna valley	Napthalene, Acenapthene, Fluorene, Phenanthrene, Anthracene, Fluoranthene, Pyrene, Benzo [a]anthracene, Chrysene, Benzo[b]fluoranthene, Benzo[k]fluoranthene, Benzo[a]pyrene, Dibenzo[a,h]anthracene, Benzo[ghi] perylene, Indeno [1,2,3-cd]pyrene	snow	mean ∑PAH: 32	[256]

**Table 7:** Acronyms reported along the text, with relative explanations.

Acronym	Meaning
ADONA	4,8-Dioxa-3H-perfluorononanoic acid
BFRs	Brominated flame retardants
BPA	Bisphenol A
CECs/ECs	Contaminants of emerging concern/emerging contaminants
CFRs	Chlorinated flame retardants
Cl-PFESA	Chlorinated perfluoroethyl sulfonate
CUPs	Currently used pesticides
DDTs	Dichlorodiphenyltrichloroethane
DIE	Dieldrin
EDCs	Endocrine disruptive chemicals
END	Endrin
EPA	Environmental Protection Agency
EU	European Union
FBSA	Perfluorobutane sulfonamide
FOSA	Perfluorooctane sulfonamide
FTSA	Fluoroteromer sulfonate acids
FTUCA	Fluoroteromer unsaturated carboxylic acids
HCH	Hexachlorocyclohexanes
HEPT	Heptachlor
HFPO-DA	Hexafluoropropylene oxide dimer acid
HMs/E-HMs/Re-HMs/CCH-HMs	Heavy metals/Emerging-heavy metals/Re-emerging heavy metals/Continuous concern historical heavy metals
ICBs	Industrial chemicals and by-products
LRT	Long-range transport
MeFBSA	N-methyl perfluorobutane sulfonamide
MeFOSE	N-methyl perfluorooctane sulfonamidoethanol
NBFRs/NCFRs	Novel brominated flame retardants/Novel chlorinated flame retardants
OCDD	Octachlorodibenzo-p-dioxin
OCPs	Organochlorine pesticides
OPEs	Organophospate esters
PAHs	Polycyclic Aromatic Hydrocarbons
PBDEs	Polybrominated diphenyl ethers
PCDD/Fs	polychlorinated dibenzo-p-dioxins/Furans
PCPs	Personal Care Products
PFAS	Poly- and perfluoroalkyl substances
PFBS	Perfluorobutane sulfonate
PFECHS	Perfluoroethylcyclohexane sulfonate
PFHxA	Perfluorohexanoic acid
PFHxS	Perfluorohexane sulfonate
PFNA	Perfluorononanoic acid
PFOA	Perfluorooctanoic acids
PFOS	Perfluorooctane sulfonic acids
POPs	Persistent organic pollutants
PPCPs	Pharmaceuticals and personal care products
SCCPs/MCCPs/LCCPs	Short chain/Mid chain/Long chain chlorinated paraffins
TC	Trans-chlordane
TCE	Technological critical element
uPCBs/PCBs	Unintentionally produced polychlorinated biphenyls/polychlorinated biphenyls
WHO	World Health Organization

## Data Availability

No new data were created or analyzed in this study. Data sharing is not applicable to this article.
